# Identification and validation of biomarkers associated with lactic acid metabolism in diabetic nephropathy

**DOI:** 10.7717/peerj.20761

**Published:** 2026-03-03

**Authors:** Hua Guo, Xiaoman Lu, Guilin Fang, Yun Qi, Huili Cui, Xiaojuan Zhang

**Affiliations:** 1 Department of Geriatric Cardiology, Jinling Hospital, Affiliated Hospital of Medical School, Nanjing University, Nanjing, Jiangsu, China; 2 National Clinical Research Center for Kidney Diseases, Jinling Hospital, Affiliated Hospital of Medical School, Nanjing University, Nanjing, Jiangsu, China

**Keywords:** Diabetic nephropathy, Lactic acid metabolism, Machine learning, Biomarkers

## Abstract

**Background:**

Previous studies have demonstrated a close association between diabetic nephropathy (DN) and lactic acid metabolism; however, the underlying mechanisms remain unclear. This study aimed to investigate the role of lactic acid metabolism-related biomarkers in the pathogenesis of DN.

**Methods:**

The DN training and validation datasets were obtained from public databases, while lactic acid metabolism-related genes (LRGs) were sourced from the literature. Using a comprehensive bioinformatics approach, we screened for potential biomarkers. Subsequent analyses included nomogram construction, functional enrichment, immune cell infiltration profiling, regulatory network mapping, drug target prediction, and molecular docking to elucidate the biomarkers’ roles in DN pathogenesis. Finally, reverse transcription quantitative polymerase chain reaction (RT-qPCR) was performed to validate biomarker expression levels in clinical samples.

**Results:**

Through a comprehensive analysis of bioinformatics methods, we identified two biomarkers—PTGS2 and NFE2L2—as significant candidates in DN. The nomogram demonstrated robust predictive efficacy, validating their utility. NFE2L2 and PTGS2 were positively correlated with the five signal pathways, such as hypoxia and IL2 STAT5 signaling. Both PTGS2 and NFE2L2 had the highest positive correlation with T follicular helper cells (correlation coefficient (cor) = 0.49, *p* < 0.01, and cor = 0.54, *p* < 0.01). Two biomarkers predicted multiple miRNAs and transcription factors (TFs), such as miR-144-3p, GATA2, and GATA3. Drug-target analysis highlighted high-affinity interactions for NFE2L2 -lagascatriol and PTGS2 -cimicoxib, further supported by molecular docking. Finally, RT-qPCR confirmed significantly elevated expression of PTGS2 and NFE2L2 in DN samples compared to controls (*p* < 0.05), aligning with bioinformatics predictions.

## Introduction

Diabetic nephropathy (DN) is a severe complication of diabetes, affecting 30–50% of patients (Alicic & Nicholas, 2022; Selby & Taal, 2020). The rising global diabetes epidemic has increased DN prevalence, straining healthcare systems (Sagoo & Gnudi, 2020). Early DN presents as glomerular hyperfiltration and microalbuminuria (>300 mg/day or >200 μg/min), progressing to macroalbuminuria, glomerular damage, and end-stage renal disease (ESRD) (Hu et al., 2023; Jin et al., 2023; Lu et al., 2020). Chronic hyperglycemia drives microangiopathy, making ESRD irreversible despite early-stage reversibility of microalbuminuria (Chen et al., 2022). Current therapies (renin-angiotensin inhibition, blood pressure, and glycemic control) show variable efficacy, necessitating better biomarkers for diagnosis and treatment (Alicic & Nicholas, 2022). Hence, investigating novel diagnostic biomarkers represents a valuable research endeavor, one that holds the potential to refine diagnostic accuracy and uncover novel therapeutic targets.

Cellular catabolism of glucose proceeds primarily through two pathways: fermentation and respiration. Fermentation, which serves as the sole metabolic option under anaerobic conditions, results in the production and excretion of lactate (Azushima et al., 2023). This process is characterized by an imbalance between ATP supply and demand, coupled with an impairment in mitochondrial electron clearance. Impaired mitochondrial function in insulin-resistant cells contributes to DN (Sharma et al., 2013). Concurrently, experimental studies have confirmed that the onset of diabetes mellitus is associated with a reduction in mitochondrial content and electron transport activity within the diabetic kidney (Chowdhury, Smith & Fernyhough, 2013). Additionally, elevated urinary lactate correlates with albuminuria and poor renal outcomes (Dugan et al., 2013), while insulin deficiency/resistance increases post-exercise muscle lactate (Darshi et al., 2024).

In summary, the pathogenesis of DN is complex and multifactorial, with lactate metabolism representing a specifically relevant component. However, the precise mechanistic links between lactate metabolism and DN remain incompletely understood. This study analyzes DN sequencing data to identify lactate metabolism-related biomarkers, elucidating their pathological mechanisms. The findings may improve DN diagnosis and personalized therapy.

## Materials & Methods

### Data sources

The GSE142153 (platform: GPL6480) and GSE154881 (platform: GPL24676) datasets were retrieved using Gene Expression Omnibus (GEO) databases, which are accessed through https://www.ncbi.nlm.nih.gov/geo/. The GSE142153 dataset was used as a training set, including 23 peripheral blood mononuclear cells (PBMCs) samples from DN patients and 10 healthy controls. The GSE154881 dataset was used as a validation set, which included four blood samples from DN patients and five from healthy controls. Furthermore, a total of 2,139 lactate metabolism-related genes (LRGs) have been identified in the literature (Li, Qian & Bao, 2024) (Table S1). All data downloaded on September 26th, 2024.

### Identification of candidate genes

DN samples & control samples from the training set were analyzed for identifying differential expression genes (DEGs) using the limma (v 3.44.3) (Ritchie et al., 2015) package with *p* < 0.05 and |log2foldchange (FC)| > 0.5, followed by volcano plots and heatmaps for the presentation of the differential analysis results using the ggplot2 (v 3.3.2) (Gustavsson et al., 2022) package and pheatmap (v 0.7.7) (Chen et al., 2021) package, respectively. In order to obtain lactate metabolism-related differential genes, intersections were taken for DEGs and LRGs, and the obtained genes were noted as intersected genes, which were visualized by the UpSet package (v 1.4.0) (Conway, Lex & Gehlenborg, 2017). Harnessing the STRING database (https://cn.string-db.org/), a protein–protein interaction (PPI) network was constructed with a confidence level of 0.7 to analyze interacting genes. This network was further visualized and represented in the software Cytoscape (v 3.10.2) (Doncheva et al., 2019) to explore intricate protein–protein interactions. Based on the constructed PPI network, potential candidate genes were screened by the cytoHubba plug-in of the Cytoscape package. Four algorithms (Closeness, Density of Maximum Neighborhood Component (DMNC), Edge Percolated Component (EPC), and Radiality) were used to systematically identify the top 25 genes within the interconnected network. The intersection of the Venn diagram was used with the ggvenn package (v 1.7.3) (Gao, Yu & Cai, 2021) to obtain candidate genes.

### Functional enrichment analysis of candidate genes

To delve into the function and pathway of the candidate genes, a functional enrichment analysis was carried out. This involved the utilization of Gene Ontology (GO) (with an adjusted *p*-value threshold of 0.05) and Kyoto Encyclopedia of Genes and Genomes (KEGG) (also with *p*-value < 0.05), facilitated by the clusterProfiler package (version 3.16.0) (Yu et al., 2012). There are three types of GO analysis: biological process (BP), molecular function (MF), and cellular component (CC). The ggplot2 package was employed to illustrate the top five enriched terms for each category, ordered by their least significant *p*-values. Moreover, the top 10 enriched KEGG pathways were showcased, prioritized by the minimal *p*-values for visibility.

### Screening of candidate biomarkers

In this study, an integrated machine learning strategy combining LASSO regression and random forest was adopted to screen candidate biomarkers. Random seeds were set to ensure the reproducibility of the analytical process. In the training set, the glmnet package (version 4.1.8) (Li, Lu & Yin, 2022c) was employed to perform LASSO regression analysis, with the regularization parameter alpha set to 0.2 to balance the characteristics of LASSO and ridge regression. The optimal lambda value (lambda.min) and its corresponding feature genes were determined through 10-fold cross-validation. This configuration not only accommodated the characteristics of small-sample data analysis but also effectively addressed the multicollinearity issues commonly present in gene expression data. Meanwhile, the randomForest package (version 1.7.3) (Chen & Boutros, 2011) was utilized for random forest analysis, with the mtry parameter set to 1 to optimize variable selection. This algorithm, through its ensemble learning and out-of-bag error estimation mechanisms, effectively assessed feature importance and controlled the risk of overfitting under limited sample conditions. The combined application of the two algorithms leveraged the advantages of LASSO regression in feature selection and model interpretability, while also harnessing the ability of random forests to handle nonlinear relationships and complex interactions. This dual-validation mechanism enhanced the reliability of the identified biomarkers, ensuring their significance in both simple linear associations and complex network interactions. Subsequently, to identify the final candidate biomarkers, the VennDiagram package (version 1.18.0) was used to obtain the intersection of the LASSO feature genes and the Random Forest feature genes. Based on the gene expression data from both the training and validation sets, the pROC package (version 1.18.0) (Love, Huber & Anders, 2014; Robin et al., 2011) was employed to generate the Receiver Operating Characteristic curve for each candidate biomarker. An area under the curve greater than 0.7 was considered indicative of good predictive power for DN diagnosis. Concurrently, to evaluate the classification performance of the genes between the DN and control groups, a Precision-Recall curve analysis was performed. The expression data for GSE142153 (training set) and GSE154881 (validation set) were obtained from the GEO database. For each dataset, the expression values of the candidate genes were extracted, and the Precision and Recall at different thresholds were calculated. The corresponding results were generated using the pr. The curve function from the PRROC (version 1.4) (Keilwagen, Grosse & Grau, 2014) package and the integral area under the curve value were used as the performance quantification metric. Finally, visualization was carried out using the ggplot2 package in R. The area under the curve (AUC) above 0.7 was thought to be a good predictor for the diagnosis of DN. To assess the consistency of expression patterns, the limma and DESeq2 packages (version 1.42.0) (Love, Huber & Anders, 2014) were used to analyze the differences in the DN and the control groups. Those biomarkers exhibiting significant differences in expression levels and a consistent trend across sets were selected as the final biomarkers (with *p*-values < 0.05, Rank sum test).

### Correlation, GeneMANIA, and subcellular localization analysis of biomarkers

A Spearman correlation analysis among biomarkers was conducted for all samples in the training set using a psychological package (v 2.2.5) (https://cran.r-project.org/web/packages/psych/psych.pdf) with a |Correlation coefficient (cor)| above 0.3 and the *p*-values below 0.05. Through GeneMANIA (http://genemania.org/), interactions of biomarkers with other genes were explored to build a coexpression network among them. To delve deeper into the mechanism of action of biomarkers, the mRNALocater database (http://bio-bigdata.cn/mRNALocater/) accessed in August 2024 was used to predict the subcellular location of these genes.

### Construction and evaluation of diagnostic model nomogram

Biomarkers’ nomogram was constructed by the rms package (v 6.5-0) (Pan et al., 2021) to evaluate the ability of biomarkers to differentiate disease samples and to infer the probability of DN occurrence in the training set. Each gene corresponded to a single score point, and the sum of the scores for each gene corresponded to the total score. The disease probability was then predicted based on the total points. A higher total score corresponded to a higher probability of the disease. After that, the predictive power of the nomogram was confirmed using regplot (v 1.1) (https://CRAN.R-project.org/package=regplot) to draw a calibration curve. The predictive performance of the biomarkers for the diagnosis of DN was evaluated by using the pROC package to construct an ROC curve, aiming to evaluate the efficacy of the nomogram. The area under the curve (AUC) above 0.7 suggests a good biomarker effect. Finally, the clinical decision value was evaluated with decision curve analysis (DCA), which was visualized by the ggDCA package (v 1.2) (Ouyang et al., 2023) to provide a comprehensive assessment of its clinical utility.

### Enrichment analysis of biomarkers

The reference gene set “c2.cp.kegg.v11.0.symbols” was obtained from the Molecular Signatures Database (MSigDB), an online repository available at the following address: https://www.gsea-msigdb.org/gsea/msigdb. The Spearman correlation analysis between each biomarker and all the other genes was calculated, and then the related genes were sorted in descending order according to the correlation coefficient to obtain a list of relevant genes for each biomarker. Based on the gene set enrichment analysis (GSEA), we performed an enrichment analysis using the clusterProfiler package. The Benjamini–Hochberg method was used for FDR correction to control for multiple testing, and the adjusted *p*-value of less than 0.05 was used for screening.

To further explore the role of gene sets, we used the GSVA package (version 1.49.4) (Hänzelmann, Castelo & Guinney, 2013) for GSVA with the “h.all.v2024.1.Hs.symbols.gmt” reference set. The limma package was utilized to identify differences in GSVA scores across DN and control groups for different sample gene sets. These findings were visualized through the ggplot2 package by creating bar graphs. The differential expression criteria for this analysis were a t-statistic absolute value of —t—>2 and a *p*-value <0.05. A positive *t*-value indicated pathway activation in the DN group, while a negative *t*-value indicated activation in the control group. The differential expression pathway of the two groups was termed the differential HALLMARK signal pathway. Based on all samples of the training set, Spearman correlation analysis between gene sets and differential HALLMARK signaling pathways in the DN and control groups was performed using the psych package with a threshold of |cor| > 0.3 and *p* < 0.05.

### Immune infiltration assessment

To assess any distinct immune status disparities between DN and control samples, we used the ssGSEA algorithm from the GSVA package to analyze immune cell infiltration in the training dataset, involving 28 distinct immune cells (Bindea et al., 2013). Each immune cell’s infiltration score was computed, and the statistical significance was set at *p* < 0.05.

The results were graphically represented as a heatmap. Following that, the Wilcoxon test was performed to determine whether there was a significant difference in the infiltration of immune cells in both groups. Again, using the psych package, Spearman correlations were examined for differential immune cells and their associations with biomarkers, requiring a correlation coefficient threshold of |cor| > 0.3 and *p*-value < 0.05.

### Construction of molecular regulatory network

To delve into the microRNAs (miRNAs) regulatory landscape of the biomarkers, we utilized the MIRNet database (https://www.mirnet.ca/) to identify miRNAs that potentially interact with the biomarkers. Then, a complex miRNA-mRNA (biomarkers) regulatory network was constructed using the Cytoscape software platform. To investigate the involvement of transcription factors (TFs) in the progression of DN, the biomarkers were input into the Network Analysts web application (https://www.networkanalyst.ca/). As our data source for TF prediction, the JASPAR database (https://jaspar.elixir.no/) was chosen. Finally, a TF-mRNA regulatory network was generated and visualized with Cytoscape, enabling the exploration of TF regulatory dynamics. To understand the RNA-binding proteins (RBPs) regulating biomarkers during DN development, the MIRNet database was used to predict the RBPs capable of interacting with biomarkers. The RBP-biomarker regulatory network was brought to life in a graphical representation using the Cytoscape software.

### Analysis of biomarker-disease associations

In order to mine the associations between biomarkers and diseases, we employed the Comparative Toxicogenomics Database (CTD) (https://ctdbase.org/) for analyzing the types of diseases related to the biomarkers. The results were sorted by the “InferenceScore” standard, with the top 20 disease types selected for further analysis. The biomarker-disease network was visualized using Cytoscape software.

### Drug prediction and molecular docking

To search for potential drug candidates that might interact with the biomarkers for DN therapy, we leveraged the drug-gene interaction data from the Drug-Gene Interaction (DGIdb) database (https://www.dgidb.org/), which provides insights into the interplay between drugs and genes. The drugs targeting the biomarkers were determined, sorted according to their interaction score, and the top 20 drugs were selected. Next, a biomarker-drug network was constructed using Cytoscape software. The drugs that showed significant correlations with the biomarkers were identified as key drug candidates. Their molecular structures were obtained from the PubChem database (https://pubchem.ncbi.nlm.nih.gov/). Simultaneously, the protein structures associated with the biomarkers were retrieved from the Protein Data Bank (PDB) database (https://www.rcsb.org/search/advanced/structure). Subsequently, the molecular structure of the critical drugs and the biomarker proteins were investigated for potential binding interactions at a molecular level by the software tool CB-Dock (http://clab.labshare.cn/cb-dock/php/), and visualized using Pymol software (v 2.5.7) (Abu Hassan, Hanževački & Pordea 2024).

### Molecular dynamics simulations

To gain insights into the drug’s mechanism of action, this study conducted 100-ns molecular dynamics (MD) simulations using GROMACS (v 2024.4) (Lemkul, 2024) to evaluate the stability of the complex formed by the drug and biomarker, as well as the kinetic characteristics of drug binding. The simulation system was based on the AMBER14SB force field and the AMBER gaff force field, generating parameter and topology files for the protein and small molecule ligand, respectively, solvated with the TIP3P water model. A cubic water box with periodic boundary conditions was applied, ensuring a 1-nm buffer distance between the solute edge and the box boundary. Na^+^/Cl^−^ ions were added at a concentration of 0.15 mol/L to maintain the electrical neutrality of the system. The system first underwent energy minimization *via* the steepest descent method to eliminate unreasonable contacts and local stresses in the initial conformation. To obtain reasonable molecular orientations and reduce errors in subsequent dynamics simulations, two-stage pre-equilibration was performed. In the first stage, NVT equilibration (constant number of particles, volume, and temperature) was conducted by coupling the temperature to 300 K using the V-rescale thermostat with a 2-fs time step for 100 ps to stabilize the system temperature. The system was then transferred to NPT equilibration (constant number of particles, pressure, and temperature), using the same temperature coupling method and time parameters, and achieving 1 bar pressure equilibration *via* the Parrinello-Rahman barostat for 100 ps to optimize solvent density distribution (gen_seed = 12,345). During the production simulation stage, a 2-fs time step was maintained, and 100-ns unrestrained dynamics sampling was completed under constant temperature and pressure conditions, with the complex’s dynamic behavior monitored throughout. Finally, the root-mean-square deviation (RMSD) of the protein-drug complex was calculated to assess binding stability, the root-mean-square fluctuation (RMSF) of protein backbone atoms was analyzed to evaluate residue flexibility changes, the total energy fluctuations of the system were characterized to assess thermodynamic stability, and the number and occupancies of hydrogen bonds between the drug and target were statistically analyzed to quantify the binding interaction strength. In addition, the distance between the binding site of the small molecule and the amino acid residues of the protein served as a key dynamic parameter for analyzing their interaction, reflecting important information such as binding stability, interaction mechanisms, and conformational changes. Additionally, to verify the reproducibility of the results, the initial velocity seed was set to 67,890 (gen_seed = 67,890), and a 100 ns molecular dynamics simulation was repeated using the same method to further confirm the reliability of the results.

### Experimental confirmation *via* reverse transcription quantitative polymerase chain reaction technique

These biomarkers were validated using reverse transcription quantitative polymerase chain reaction (RT-qPCR). This study was collected from Jinling Hospital Affiliated to Nanjing University based on the inclusion criteria: (1) Diagnosed with type 1 or type 2 diabetes, with a disease course usually exceeding 5 years; (2) the urinary microalbumin excretion rate (UAE) remains at 30–300 mg/24 h, or the urinary albumin/creatinine ratio (UACR) is ≥30 mg/g (at least two repeated tests within 3 months are required for confirmation); (3) estimated glomerular filtration rate (eGFR) ≥60 mL/min/1.73m^2^, excluding those with significant renal function decline; (4) age between 18 and 75 years old. Exclusion criteria: (1) Other kidney diseases: Presence of clear non-diabetic nephropathy (such as primary glomerulonephritis, polycystic kidney disease, *etc*). (2) Recent acute complications of diabetes (such as ketoacidosis, hyperosmolar state) or severe infections, combined with serious cardiovascular and cerebrovascular diseases, liver dysfunction, or other systemic diseases. (3) Pregnant or lactating women. (4) Recently participated in other clinical trials or is currently using research drugs. A total of five pairs of blood samples from the subjects and five pairs from the control group were collected. All participants signed the written informed consent form. The clinical information of the samples can be found in the Table S2. This study was approved by the Clinical Research Ethics Committee of Jinling Hospital (DZQH-KYLL-24-04). Ten fresh blood samples were taken, and the PBMCs were separated by 2,000 g centrifugation for 20 min using an equal volume of PBMC separation solution (three ml when blood <three ml), which was located in the second circular milky white layer. After resuspending the PBMCs with phosphate-buffered saline (PBS), centrifugation at 1,000 g for 10 min, discarding the supernatant, adding one ml of TRIzol to lyse the cells, and the lysed samples can be frozen in a −80 °C freezer. Total RNA was extracted using TRIzol reagent (Ambion, Austin, TX, USA). During the extraction process, after adding chloroform for stratification, centrifugation at 12,000 g and 4 °C for 15 min, the upper aqueous phase was taken, an equal volume of ice isopropanol was added to precipitate RNA, and after multiple ethanol washes and drying, it was dissolved in RNase-free water. RNA concentration and purity were measured using the NanoPhotometer N50, and A260/A280 and A260/A230 ratios were recorded. If the isolated RNA is not in use, freeze it in a −80 °C freezer. The mRNA reverse transcription was performed using the SweScript First Strand cDNA synthesis kit (Servicebio, Wuhan, China). The reaction consists of 5x Reaction Buffer four ul, Primer one ul, SweScript RT I Enzyme Mix one ul, Total RNA two ug, and water to 20 ul. The reaction conditions were 25 °C for 5 min, 50 °C for 15 min, 85 °C for 5 s, and 4 °C hold. The conditions for cDNA synthesis were recorded, and specific storage conditions were not mentioned after cDNA synthesis. RT-qPCR was carried out using a Master Mix of 2 x Universal Blue SYBR Green qPCR. The PCR primer sequence is presented in Table 1. GAPDH was used as a reference gene. The qPCR reaction consists of cDNA three ul, 2xUniversal Blue SYBR Green qPCR Master Mix five ul (Servicebio, Wuhan, China), Forward primer (10 μM) one ul, and Reverse primer (10 μM) one ul. A 96-well PCR plate (Sevier, PCR-9601-NS) was used using the CFX Connect Real-Time PCR Instrument (Cat. No. XLFZ006) for 40 cycles with predenaturation at 95 °C for 1 min, denaturing at 95 °C for 20 s, annealing at 55 °C for 20 s, and extension at 72 °C for 30 s. Graphpad Prism 5 software was used for data analysis, and the relative expression of genes was calculated by the 2^−△△Ct^ method (Livak & Schmittgen, 2001) and the 2^−△△Ct^ values were calculated by △Ct = Ct (target gene)−Ct (internal reference gene), △△CT = △Ct (experimental group)−△Ct (control group). The experiment was set up with five biological replicates; the number of technical replicates was not specified, and the *P*-value of the difference between groups was calculated to assess the significance of the results.

**Table 1:** Primer sequences for RT-qPCR.

Primer	Sequences
NFE2L2 F	AGGTTGCCCACATTCCCAAA
NFE2L2 R	ACGTAGCCGAAGAAACCTCA
PTGS2 F	TTGCATTCTTTGCCCAGCAC
PTGS2 R	ACCGTAGATGCTCAGGGACT
GAPDH F	ATGGGCAGCCGTTAGGAAAG
GAPDH R	AGGAAAAGCATCACCCGGAG

Total RNA was extracted from 10 samples in accordance with the instructions given by the manufacturer (Ambion). SweScript-First-strand-cDNA-synthesis-kit (Servicebio, Wuhan, China) was applied to reverse transcription of total RNA into cDNA according to the manufacturer’s instructions. RT-qPCR was carried out using a Master Mix of 2 x Universal Blue SYBR Green qPCR (Servicebio, Wuhan, China). The PCR primer sequence is presented in Table 1. GAPDH was used as a reference gene. The 2−ΔΔCt method was used to calculate the expression of biomarkers.

### Statistical analysis

The statistical analysis was performed with the R software (v 4.3.3) (R Core Team, 2024; Fujisawa et al., 2023), and the Wilcoxon Test was used to evaluate inter-group differences, and for *p* values below 0.05 a statistical significance was considered.

## Results

### Identification and functional analysis of 21 candidate genes

**Figure 1:**
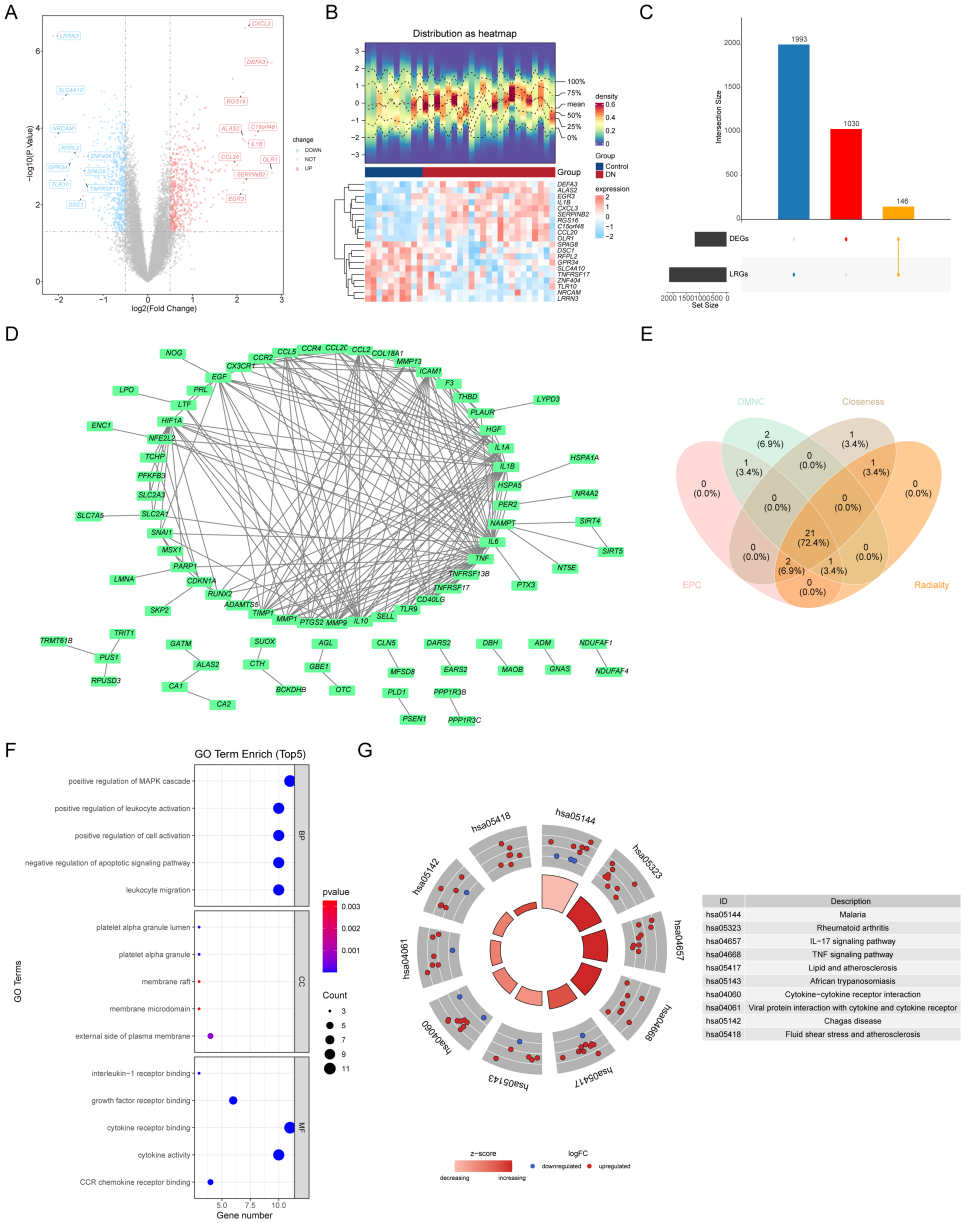
Identification and analysis of biomarkers in diabetic nephropathy (DN). (A) Volcano plot showing differentially expressed genes (DEGs) between DN and control groups. Red and blue dots represent upregulated and downregulated genes, respectively. (B) Heatmap of gene expression distribution in DN and control groups, highlighting key genes such as DEFA3, IL1B, and CXCL3. (C) Venn diagram illustrating the intersection of lactic acid metabolism-related genes (LRGs) and DEGs. (D) Protein–protein interaction (PPI) network of key genes, including NFE2L2 and PTGS2, involved in DN. (E) Network centrality analysis (Neighborhood Component (DMNC), Closeness, Edge Percolated Component (EPC), Radiality) of genes in the PPI network. (F) Top 5 enriched Gene Ontology (GO) terms in biological processes (BP), cellular components (CC), and molecular functions (MF), highlighting key pathways like MAPK cascade and cytokine activity in diabetic nephropathy. (G) Enrichment analysis of signaling pathways associated with DN. The left panel features a bar chart within a circle. The height of the bars represents the significance of the terms, with higher bars indicating greater significance. The color of the bars indicates the z-score, with darker colors corresponding to higher z-scores. The outer circle displays the expression levels of each gene in each term as a scatter plot, with red and blue representing upregulated and downregulated genes, respectively. The right panel provides descriptions of the KEGG enrichment terms.

Upon differential expression profiling in the training dataset, there were 1,176 DEGs in the DN and control groups, including 596 up-regulated and 580 down-regulated genes. These genes were featured prominently in the volcano plot and heatmap (Figs. 1A–1B), where they were arranged according to their —log2FC— values for better visualization. The top 10 upregulated and downregulated DEGs were highlighted for further scrutiny. Intersections were taken for DEGs and LRGs, and 146 intersected genes for further analyzed (Fig. 1C). The protein interactions among the intersected genes were investigated, resulting in the protein interaction network comprising 86 proteins (confidence = 0.7). Within this network, TNF, IL6, and IL1B exhibited extensive interactions with other genes (Fig. 1D). Four algorithms (Closeness, DMNC, EPC, and Radiality) were employed to identify the top 25 genes in the PPI network. The genes obtained from each algorithm were then intersected, resulting in a final set of 21 candidate genes (Fig. 1E, Table S3). Following the differential expression analysis, we performed a thorough GO and KEGG functional analysis of 21 candidate genes. The GO analysis showed a total of 1,190 classifications: 1,150 biological processes (BP), nine cellular components (CC), and 31 molecular functions (MF). Notably, the GO-BPs disclosed a significant enrichment in negatively regulated apoptotic signaling pathway (GO: 2001234) and positive regulation of mitogen-activated protein kinase cascade (GO: 0043410). In the GO-CCs, platelet alpha granule lumen (GO: 0031093) and platelet alpha granule (GO: 0031091) stood out. The GO-MFs demonstrated enrichment in cytokine receptor binding (GO: 0005126) and cytokine activity (GO: 0005125). This information is depicted in Fig. 1F and further detailed in Table S4. Additionally, KEGG analysis revealed enrichment in 131 functional pathways, with them such as malaria (hsa05144), rheumatoid arthritis (hsa05323), and IL-17 signaling pathway (hsa04657) (Fig. 1G, Table S5).

### Identification of *NFE2L2* and *PTGS2* as biomarkers

**Figure 2:**
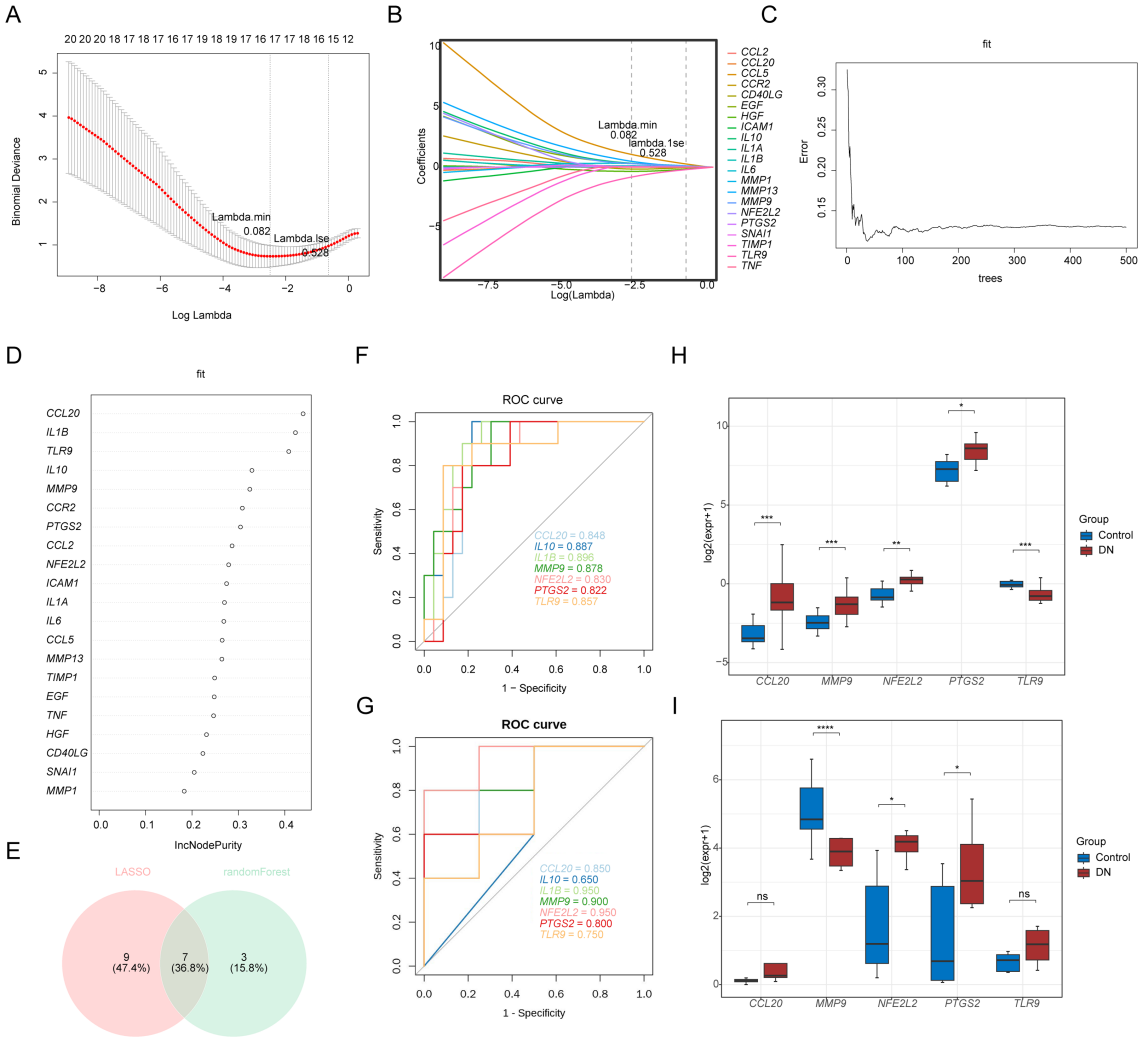
Biomarker selection, validation, and diagnostic performance in diabetic nephropathy (DN). (A) Least absolute shrinkage and selection operator (LASSO) coefficient profile plot. The *x*-axis represents the logarithm of lambdas, and the *y*-axis represents the variable coefficients, with each line corresponding to a gene. (B) Ten-fold cross-validation for parameter tuning in LASSO analysis. The *x*-axis represents the logarithm of lambdas, and the *y*-axis represents the model error. In the figure, lambda.min refers to the regularization parameter value that minimized the model prediction error during the 10 - fold cross - validation process. Lambda.1se refers to the regularization parameter value obtained by adding one standard error (se) to the minimum error corresponding to lambda.min. (C) RandomForest screening results. The *y*-axis represents genes, and the *x*-axis represents the IncNodePurity index. (D) RandomForest cross-validation curve showing the relationship between model error (*y*-axis) and the number of trees used for fitting (*x*-axis). (E) Venn diagram showing the intersection of genes selected by LASSO and Random Forest. Receiver operating characteristic (ROC) Curve Analysis of Candidate Biomarkers in the Training Set (F) and Validation Set (G). The *x*-axis (1-Specificity, False Positive Rate (FPR)) represents the false positive rate, and the *y*-axis (Sensitivity, True Positive Rate (TPR)) represents the true positive rate. The ROC curve reflects the relationship between sensitivity and specificity. The closer the *x*-axis is to zero, the higher the accuracy; the larger the *y*-axis, the better the accuracy. Evaluation of Candidate Biomarker Expression in the Training Set (GSE145725) (H) and Validation Set (GSE44270) (I). The *x*-axis of the boxplot represents genes, and the *y*-axis represents expression levels. Red indicates DN samples, and blue indicates control samples. Asterisks denote significance: *p < 0.05, **p < 0.01, ***p < 0.001, ****p < 0.0001, and ns indicates no significance.

In the LASSO regression analysis, lambda.min was 0.082, from which 16 LASSO feature genes were screened, including *IL6*, *IL1B*, *IL10*, *IL1A*, *MMP9*, *CCL5*, *EGF*, *MMP1*, *PTGS2*, *CD40LG*, *CCL20*, *TLR9*, *HGF*, *MMP13*, *NFE2L2*, and *SNAI1* (Figs. 2A–2B). Next, Random Forest analysis was conducted employing these candidate genes as input features, with the parameter settings of mtry = 1. The top 10 genes designated as feature genes 2 included *CCL20*, *IL1B*, *TLR9*, *IL10*, *MMP9*, *CCR2*, *PTGS2*, *CCL2*, *NFE2L2*, and *ICAM1* (Figs. 2C–2D). The intersection of genes selected by the LASSO and Random Forest algorithms resulted in seven genes, which were considered as candidate biomarkers (Fig. 2E). The results showed that with the exception of IL10, all genes exhibited AUC values greater than 0.7 in both the training set (Fig. 2F) and validation set (Fig. 2G). The precision–recall curves also demonstrated AUC values exceeding 0.7, indicating favorable precision and recall performance of the model (Fig. S1). Consequently, the final candidate biomarkers consisted of *CCL20*, *IL1B*, *MMP9*, *NFE2L2*, *PTGS2*, and *TLR9*. Due to the lack of data for *IL1B* in the validation set, it was excluded from the expression difference analysis. In both the training and validation cohorts, *NFE2L2* and *PTGS2* showed a significantly lower expression level in the control than in the DN sample, with *p* values below 0.05, as illustrated in Fig. 2H for the training set and Fig. 2I for the validation set. These were recorded as biomarkers for further analysis.

### Correlation, GeneMANIA, and subcellular localization results for biomarkers

**Figure 3:**
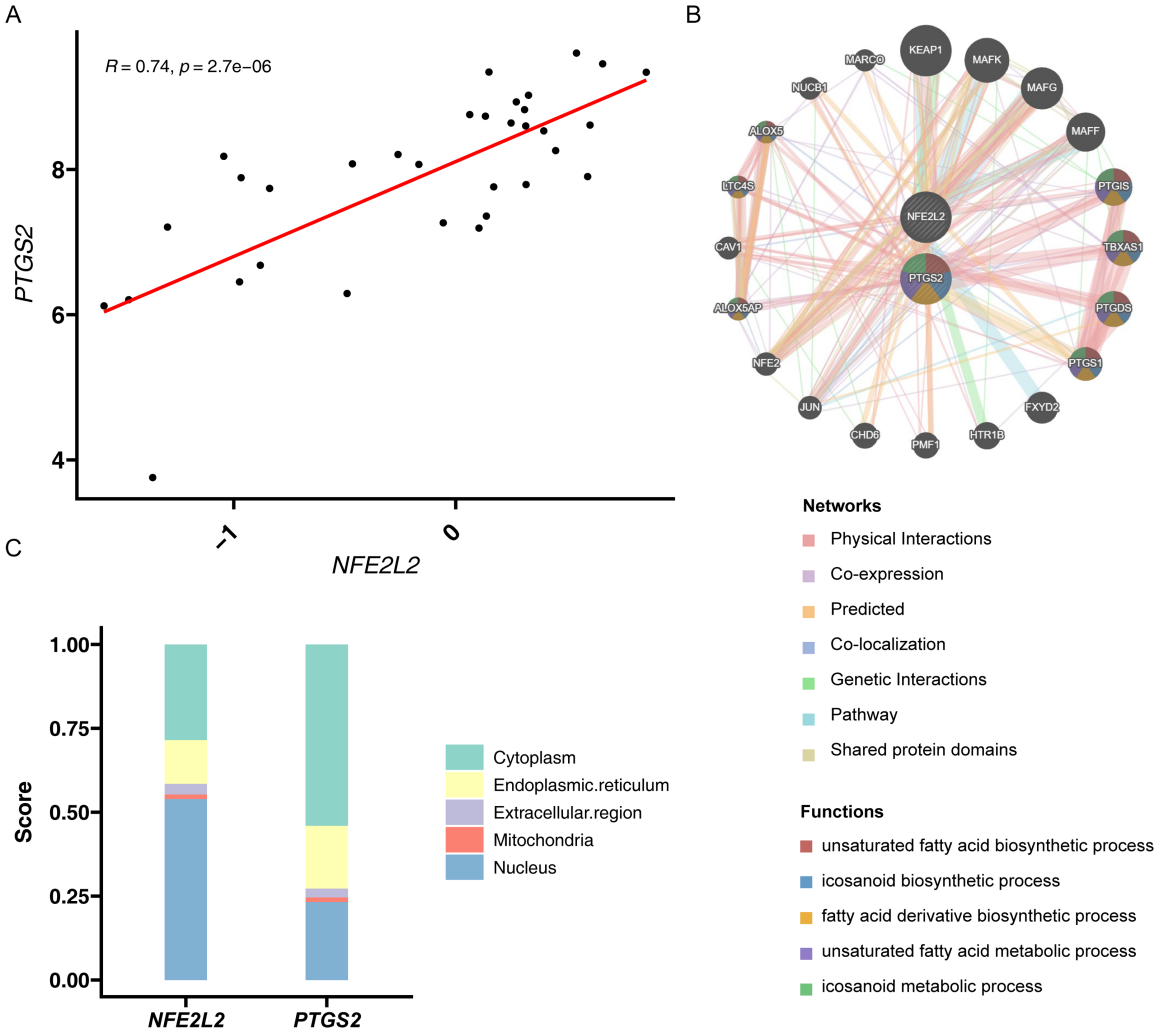
Functional and interaction analysis of *PTGS2* and *NFE2L2* in diabetic nephropathy (DN). (A) Correlation analysis between *PTGS2* and *NFE2L2* expression levels, the horizontal coordinate is the expression of *PTGS2* and the vertical coordinate is the expression of *NFE2L2*. (B) GeneMANIA network analysis illustrating the functional interactions of *PTGS2* and *NFE2L2* with other genes, including physical interactions, co-expression, and shared pathways, different coloured lines represent different networks. (C) Subcellular localization analysis of *PTGS2* and *NFE2L2*.

A Spearman’s correlation analysis showed that *NFE2L2* was positively correlated with *PTGS2*. The correlation coefficient was 0.74, and the high statistical significance was 2.7e−06, depicted in Fig. 3A. GeneMANIA analysis showed 20 functional genes related to *NFE2L2* and *PTGS2*. *NFE2L2* interacted with *KEAP1* and *MAFK*, and *PTGS2* interacted with *PTGS1* and *TBXAS1*. The analysis indicated a functional link between the two biomarkers, suggesting their involvement in processes such as unsaturated fatty acid biosynthesis, icosanoid biosynthesis, and fatty acid derivative biosynthesis (Fig. 3B). Subcellular localization further clarified that *NFE2L2* predominantly localized to the nucleus, whereas *PTGS2* was primarily found in the cytoplasm (Fig. 3C).

### *NFE2L2* and *PTGS2* as biomarkers with good diagnostic performance

**Figure 4:**
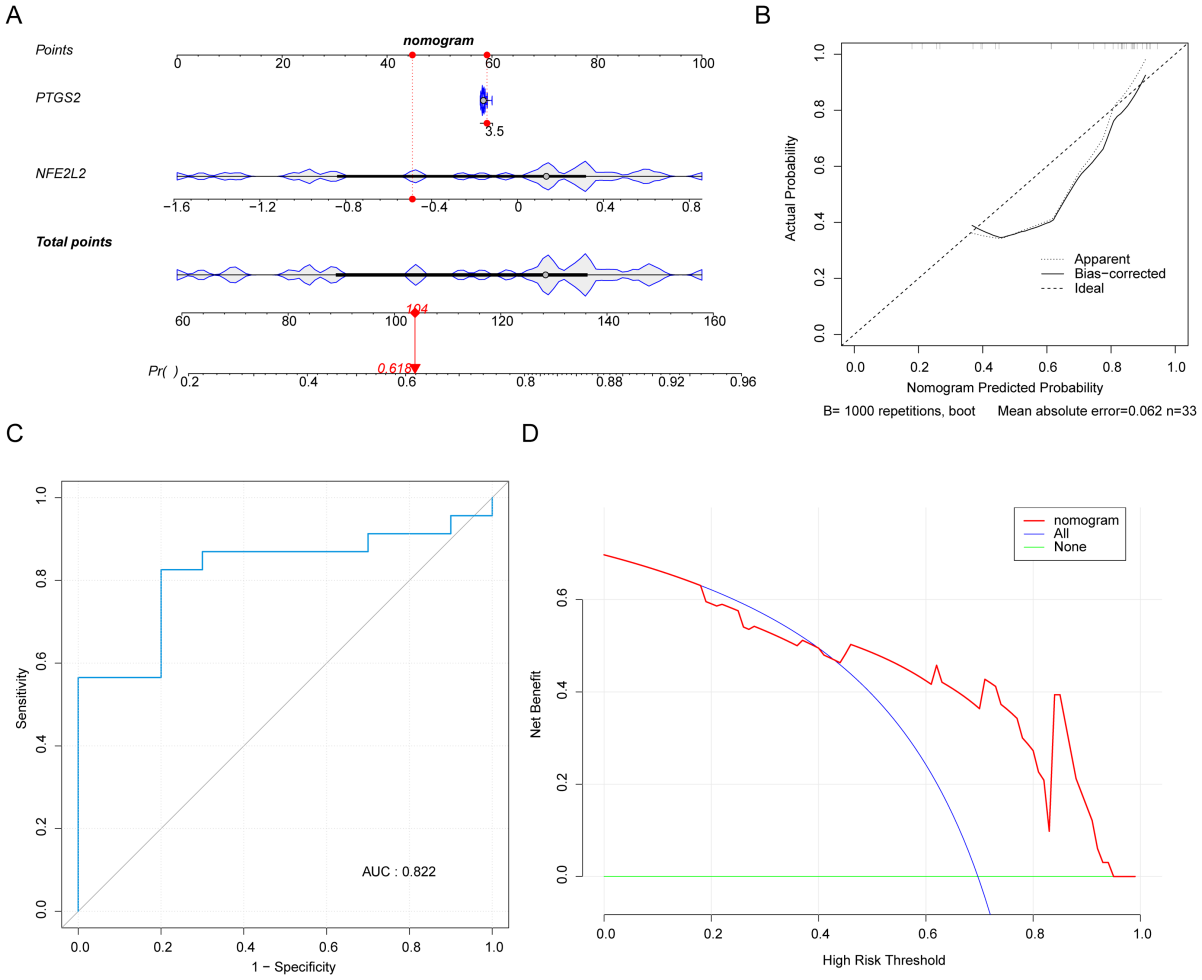
Nomogram and predictive performance of *PTGS2* and *NFE2L2* in diabetic nephropathy (DN). (A) Nomogram for predicting DN risk based on *PTGS2* and *NFE2L2* expression levels. Points are assigned to each biomarker, and the total points correspond to the predicted probability of DN. (B) Horizontal coordinate indicates the model prediction probability, vertical coordinate indicates the actual probability, Ideal line indicates that the model prediction is exactly the same as the actual in the ideal case, Bias-corrected solid line indicates the model performance when the sample is trained by repeated sampling, and Nonparametric is the performance of the training set GSE142153. (C) The receiver operating characteristic (ROC) curve , the larger the area under the curve (AUC) the better, with 0.7 as the minimum standard. (D) *X*-axis represents the risk thresholds, and *Y*-axis is the net gain; None straight line represents the line of net gain when all subjects are not intervening, and since there is no intervention, there are no true positives and false positives, and the net gain is 0; all curve is the net gain on each risk threshold when all subjects are intervening, and the slope is negative, and its intersection with the None line is the prevalence calculated from the dataset. nomogram curves are the net gain at the risk thresholds based on the risk probabilities estimated from the prediction model developed.

In the nomogram, when a single sample was randomly selected, the probability of having DN was 0.618 when the total score of two biomarkers was 104 (Fig. 4A). Moreover, the calibration plot shows that the biomarker-based model is superior in the diagnosis of DN (Fig. 4B). The AUC derived from the ROC curve was 0.822, confirming that the model has excellent predictive precision (Fig. 4C). Based on the decision curve analysis, this model obtained the benefit within the 0–1 high-risk range, and the overall clinical benefit was greater than the diagonal (Fig. 4D). Further verified the effectiveness of our model.

### Correlations between biomarkers and GSEA enrichment pathway genes

**Figure 5:**
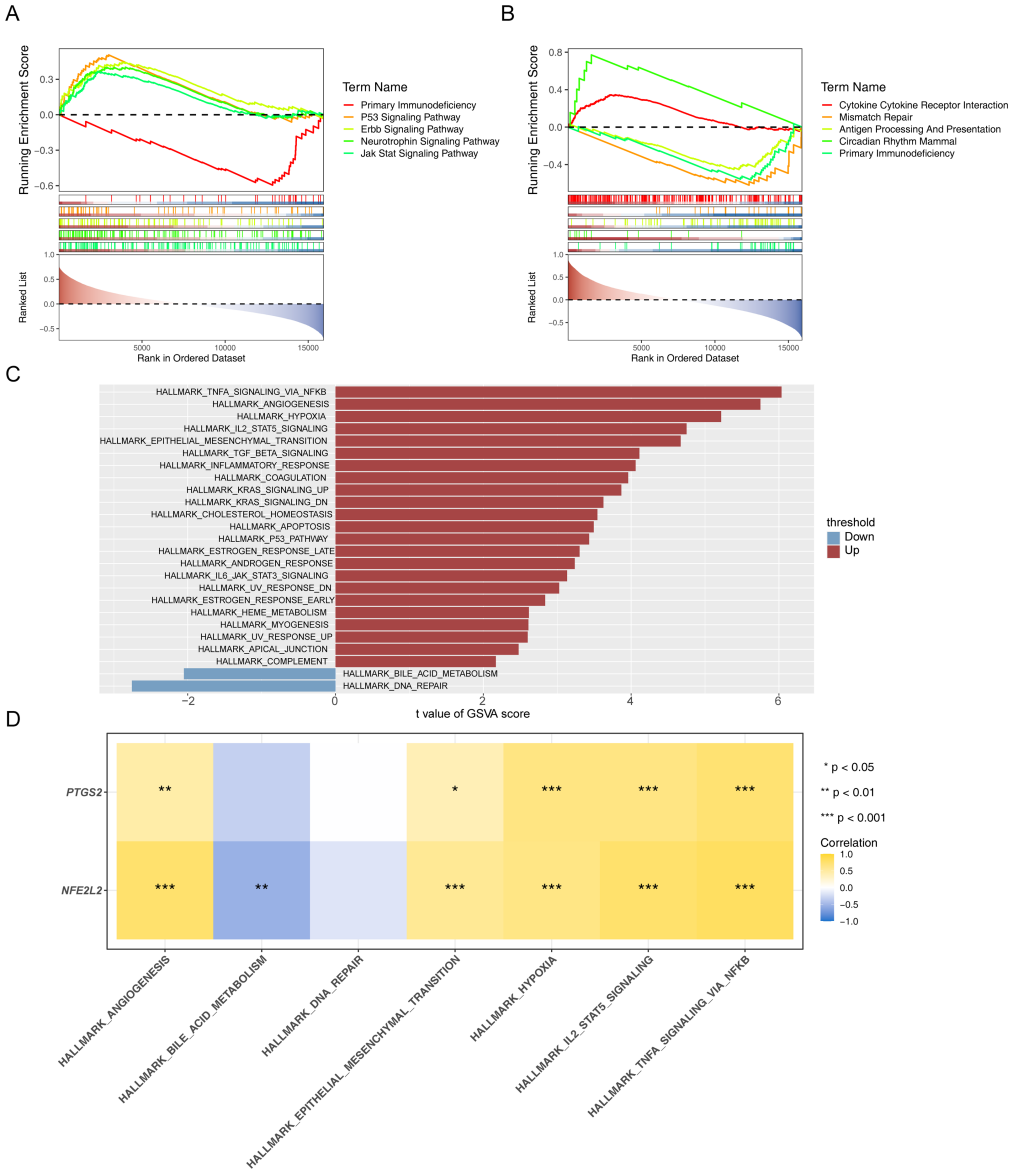
Gene set enrichment analysis (GSEA) and gene set variation analysis (GSVA) results. (A) Results for KEGG enrichment of PTGS2 single gene GSEA in training set (top5). (B) Results of KEGG enrichment of NFE2L2 single gene GSEA in the training set (top5). (C) GSVA functional enrichment analysis. (D) Correlation analysis between gene sets and differential HALLMARK signalling pathways in various samples in both DN *vs* Control groups.

The GSEA uncovered a prominent enrichment of genes linked to *PTGS2* in 17 distinct KEGG pathways, including primary immunodeficiency, P53 signaling pathway, and Erbb signaling pathway, as detailed in Table S6. The results only displayed the top five pathways (Fig. 5A). While those linked to the *NFE2L2* were enriched in 22 KEGG pathways, such as antigen processing and presentation, mismatch repair, and cytokine receptor interaction (Table S7). The results only displayed the top five pathways (Fig. 5B). Comparison of GSVA scores of gene sets between DN and control groups across different samples, the analysis results showed that there were a total of 25 pathways with significant differences between the two groups in the training set (Fig. 5C). Denoted as differential HALLMARK signal pathways. Spearman correlation analysis was conducted between gene sets and differential HALLMARK signaling pathways in various samples of DN *vs* Control groups, with an correlation absolute cor value greater than 0.3 and a *p*-value less than 0.05.) (Fig. 5D). The results showed that *NFE2L2* and *PTGS2* are positively correlated with five HALLMARK signal pathways, such as hypoxia and IL2 STAT5 signaling. *NFE2L2* is negatively correlated with HALLMARK bile acid metabolism.

**Figure 6:**
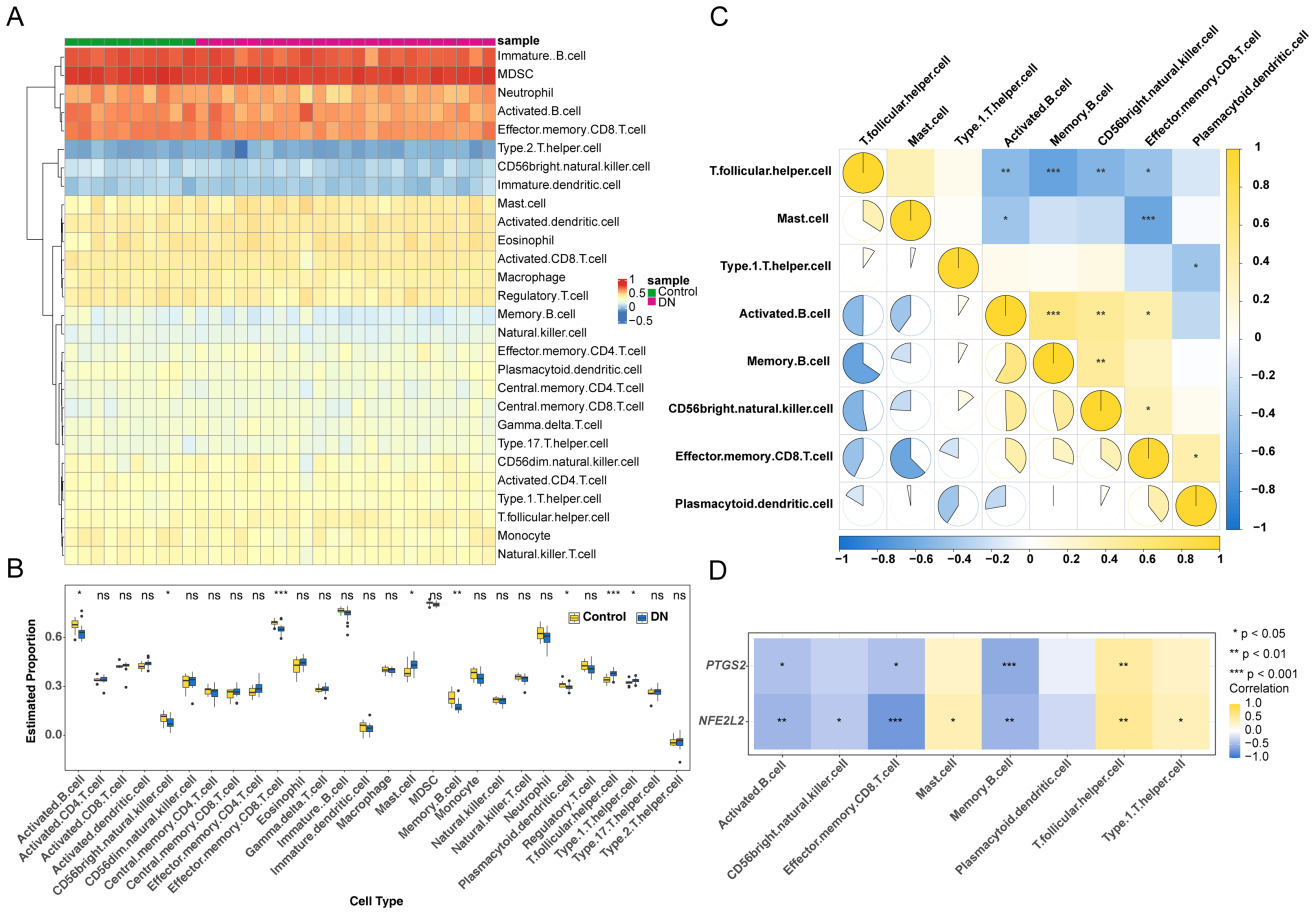
Correlation analysis of immune cell types and gene expression. (A) Heatmap of different cell contents calculated by ssGSEA; each cell in the figure represents the cell content in the sample. The top row shows the groupings of the training set. Each row represents a type of cell, and each column represents a sample. The redder the color, the higher the cell content. (B) Expression of immune infiltrating cells in DN *vs* Control samples. Asterisks indicate significance: * *p* < 0.05; ** *p* < 0.01; *** *p* < 0.001. (C) Correlation analysis of differential immune cells; the horizontal and vertical axes in the figure represent differential immune cells. The colors in the legend represent the enrichment correlation coefficient, with yellow indicating positive correlation and blue indicating negative correlation. (D) Correlation analysis of differential immune cells and biomarkers;the color changes from yellow to blue in the figure indicate the correlation changes from positive to negative.

### Biomarkers significantly correlate with immune-infiltrating cells

Gene expression data from the complete set of training samples were subjected to ssGSEA in order to perform immune cell infiltration assessment. The enrichment scores of 28 immune cell types were calculated, and the sample exhibited the highest scores for immature B cells (Fig. 6A). Then, the difference analysis of immune cells between the two groups was carried out, with *p* < 0.05 as the threshold, and eight kinds of immune infiltrating cell enrichment scores with significant differences between the groups (Fig. 6B). Among them, CD8 effect memory T cells showed the most significant difference in infiltration abundance in DN and control samples. A Spearman’s rank correlation analysis showed that there was a significantly positive correlation (cor = 0.58, *p* < 0.05) between memory B cells and activated B cells, and a strong negative correlation (cor = −0.65, *p* < 0.05) between T follicular helper cells and memory B cells (Fig. 6C, Table S8). The biomarkers, *PTGS2* and *NFE2L2*, displayed distinct patterns: *PTGS2* correlated positively with T follicular helper cells (cor = 0.49, *p* = 0.0042) and negatively with memory B cells (cor = −0.59, *p* = 0.0003), while *NFE2L2* was most positively correlated with T follicular helper cells (cor = 0.54, *p* = 0.0011) and negatively correlated with effector memory CD8 T cells (cor = −0.72, *p* < 0.0001) (Fig. 6D). In summary, the study investigated correlations between immune cell subpopulations and two biomarkers, revealing differential patterns associated with distinct immune response dynamics.

### Molecular regulatory network of *NFE2L2* and *PTGS2*

**Figure 7:**
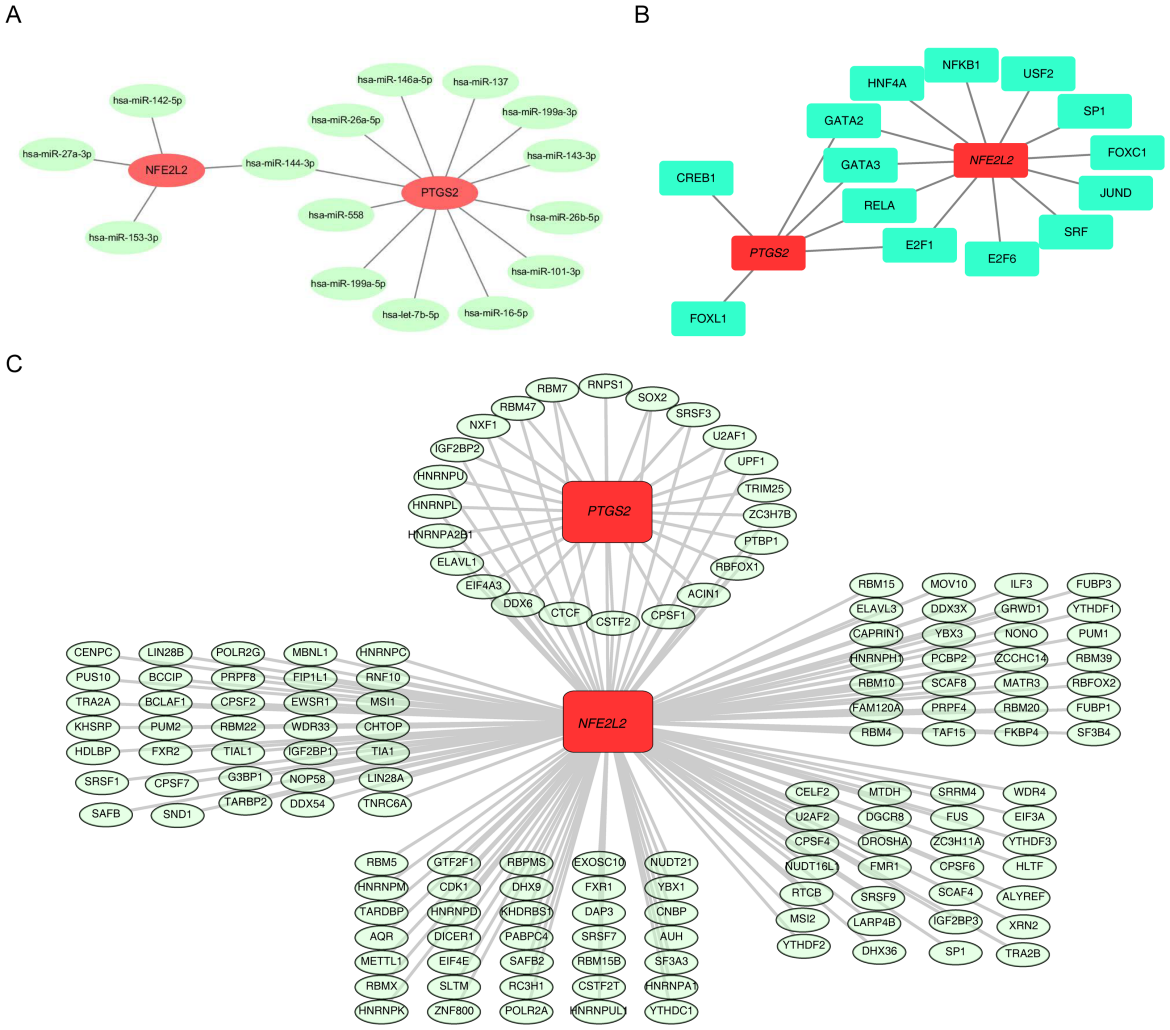
Construction of molecular regulatory network. (A) miRNA-mRNA (biomarker) regulatory network; red represents mRNA (biomarker); green represents miRNA. (B) RBP-mRNA (Biomarker) regulatory network; red represents mRNA (biomarker); green represents TF (transcription factor). (C) RBP-mRNA (Biomarker) Regulatory Network; red squares represent mRNA (biomarker); green ellipses represent RBP (RNA-binding protein).

*NFE2L2* was predicted to have four miRNAs in the MIRNet database. For *PTGS2*, MIRNet had 12 miRNAs, resulting in one intersected miRNA: miR-144-3p (Fig. 7A, Table S9). In addition, *NFE2L2* was predicted to have 12 TFs in the JASPAR database. *PTGS2* is predicted to have six TFs, resulting in four intersected TFs: GATA2, GATA3, RELA, and E2F1 (Fig. 7B). The MIRNet database predicted the RBPs that could interact with the biomarkers, and the analysis showed that *NFE2L2* predicted 148 RBPs and *PTGS2* predicted 23 RBPs. The intersection of the two biomarkers predicted resulted in 23 RBPs in common, such as CTCF, NXF1, and SOX2 (Fig. 7C, Table S10). These interactions indicate that both *NFE2L2* and *PTGS2* are regulated by a shared set of transcriptional and post-transcriptional regulators, such as CTCF, NXF1, and SOX2, highlighting potential cross-talk in molecular networks that contribute to common biological processes like gene expression regulation and signaling pathways. These findings contribute to understanding the complex regulatory dynamics of these biomarkers in cellular pathways.

### Associated diseases, drug prediction, and molecular docking of *NFE2L2* and *PTGS2*

**Figure 8:**
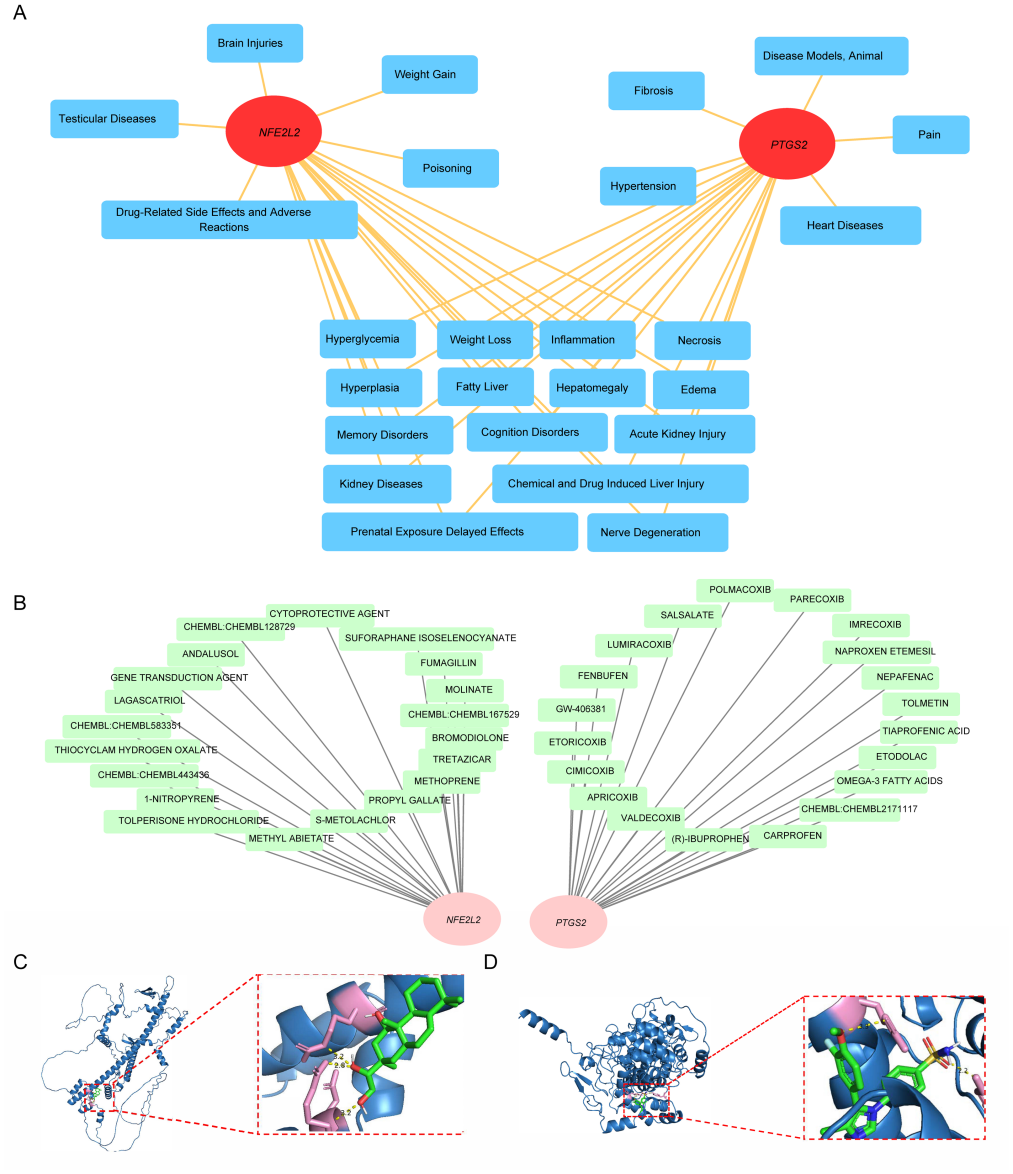
Comprehensive analysis of chemical agents and their associated health effects. (A) Biomarkers and regulatory networks of top 20 disease types; the red ovals are biomarkers and the blue squares are regulatory networks for the top 20 disease types. (B) Biomarkers and regulatory network of top 20 drug types;the pink ovals are biomarkers and the green squares are regulatory networks for the top 20 disease types. (C) Molecular docking results of *NFE2L2*-LAGASCATRIOL; the blue spiral structure is the receptor protein, the green stick model is the active molecule LAGASCATRIOL, and the nearby yellow dashed line is the hydrogen bond formed between the active ingredient and the amino acid residue. (D) Results of *PTGS2*-CIMICOXIB molecular docking; the blue spiral structure is the receptor protein, the green stick model is the active molecule CIMICOXIB, and the yellow dotted line near the active ingredient is the hydrogen bond formed between the amino acid residue.

In the CTD database, there were 2,768 disease types related to *PTGS2* and 2,666 disease types related to *NFE2L2* (Tables S11, S12). Among the top 20 disease types predicted by *NFE2L2* and *PTGS2*, there were 15 disease types in common, such as hyperglycemia, inflammation, and kidney diseases (Fig. 8A). The drug prediction results indicated that the top 20 drugs with the highest interaction scores of *PTGS2* and *NFE2L2*, respectively (Fig. 8B). In this condition, no drugs were related to drugs that they shared. Drugs with the highest interaction score were recorded as key drugs, and subsequent molecular docking was performed (Table S13). Docking results showed that the free binding energy of *NFE2L2*-lagascatriol was −7.8 kcal/mol (Fig. 8C) and that of *PTGS2*-cimicoxib was −7.3 kcal/mol (Fig. 8D), both of which were lower than -5 kcal/mol. It suggests a potent affinity between the selected drugs and their respective targets. In summary, the docking studies confirmed the high binding affinity between these drug candidates and their molecular targets.

### Molecular dynamics validation

**Figure 9:**
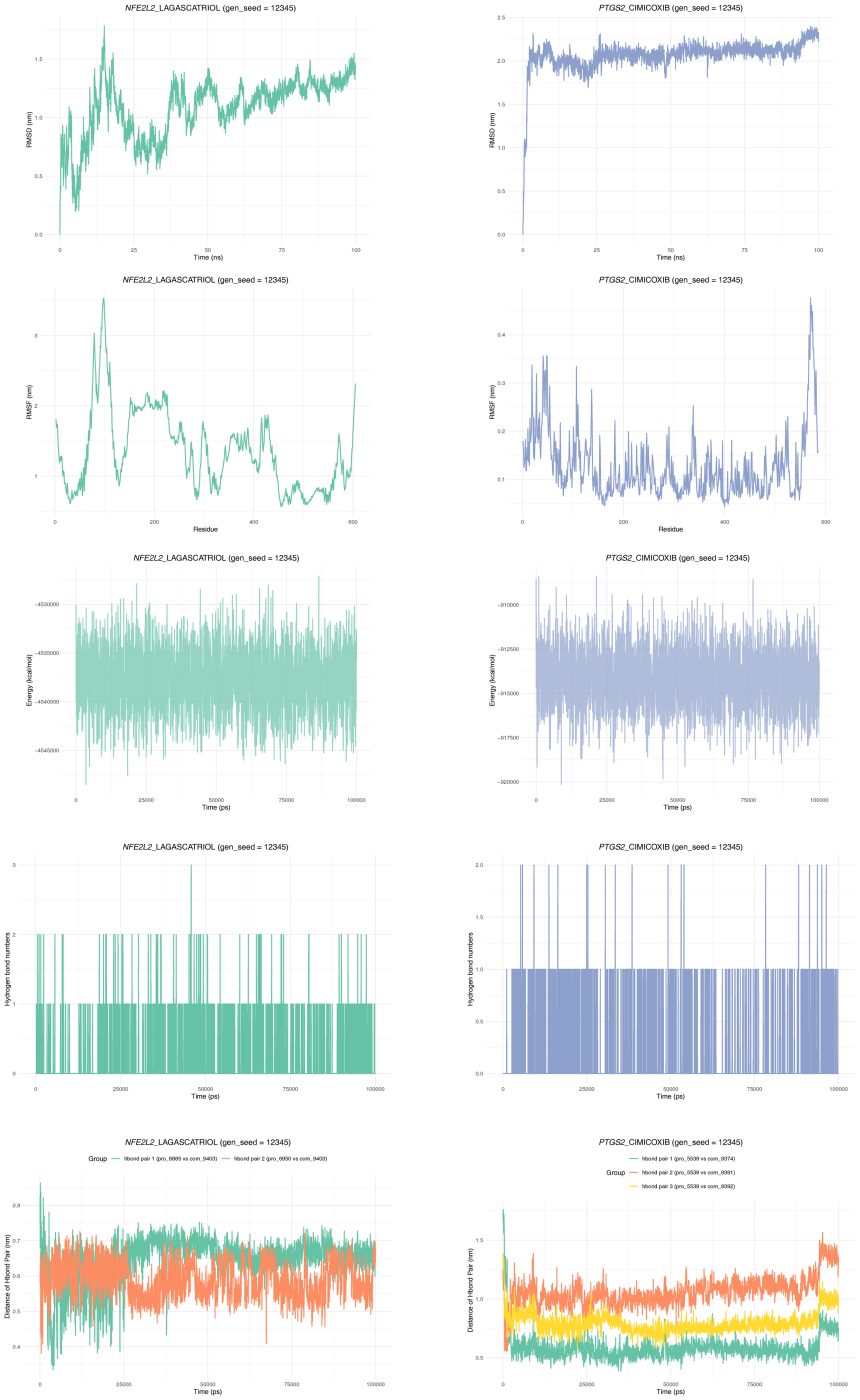
Molecular dynamics simulation of the *NFE2L2*-LAGASCATRIOL and *PTGS2*-CIMICOXIB complexes with gen_seed = 12,345. The detailed results of the molecular dynamics simulation of the NFE2L2-LAGASCATRIOL and PTGS2-CIMICOXIB complexes, successively presenting the changes in RMSD, RMSF, energy, the number of hydrogen bonds, the changes in the distance between atoms of different hydrogen bond pairs over time, and the changes in the distance between atoms of different hydrogen bond pairs over time.

This study conducted a 100-nanosecond molecular dynamics simulation on the complex of *NFE2L2*-LAGASCATRIOL and *PTGS2*-CIMICOXIB (gen_seed = 12,345) (Fig. 9). Results showed that the RMSD of the *NFE2L2*-LAGASCATRIOL complex displayed “significant early flexibility followed by relative stability, and the *PTGS2*-CIMICOXIB complex entered stable dynamic equilibrium directly after initial conformational adjustment, indicating stable ligand binding. RMSF analysis revealed *NFE2L2*-LAGASCATRIOL had” flexible hotspots at the N-terminus and partial mid-region, with rigid C-terminus and core domains. *PTGS2*-CIMICOXIB remained overall stable with localized high-flexibility residues. Total energy remained both complexes maintained balanced energy fluctuations, reflecting thermodynamic stability. Hydrogen bond analysis showed *NFE2L2*-LAGASCATRIOL formed 1–2 hydrogen bonds in dynamic equilibrium. *PTGS2*-CIMICOXIB predominantly formed 1 stable hydrogen bond. Hydrogen bond distances showed *NFE2L2*-LAGASCATRIOL’s two hydrogen bond pairs (pro_6885 *vs* com_9403; pro_6950 *vs* com_9403) fluctuated within 0.4–0.8 nm. *PTGS2*-CIMICOXIB’s three pairs (pro_5539 *vs* com_9374/9391/9392) showed mixed stability, with two pairs fluctuating up to 1.5 nm and one remaining stable (0.5–0.8 nm).

**Figure 10:**
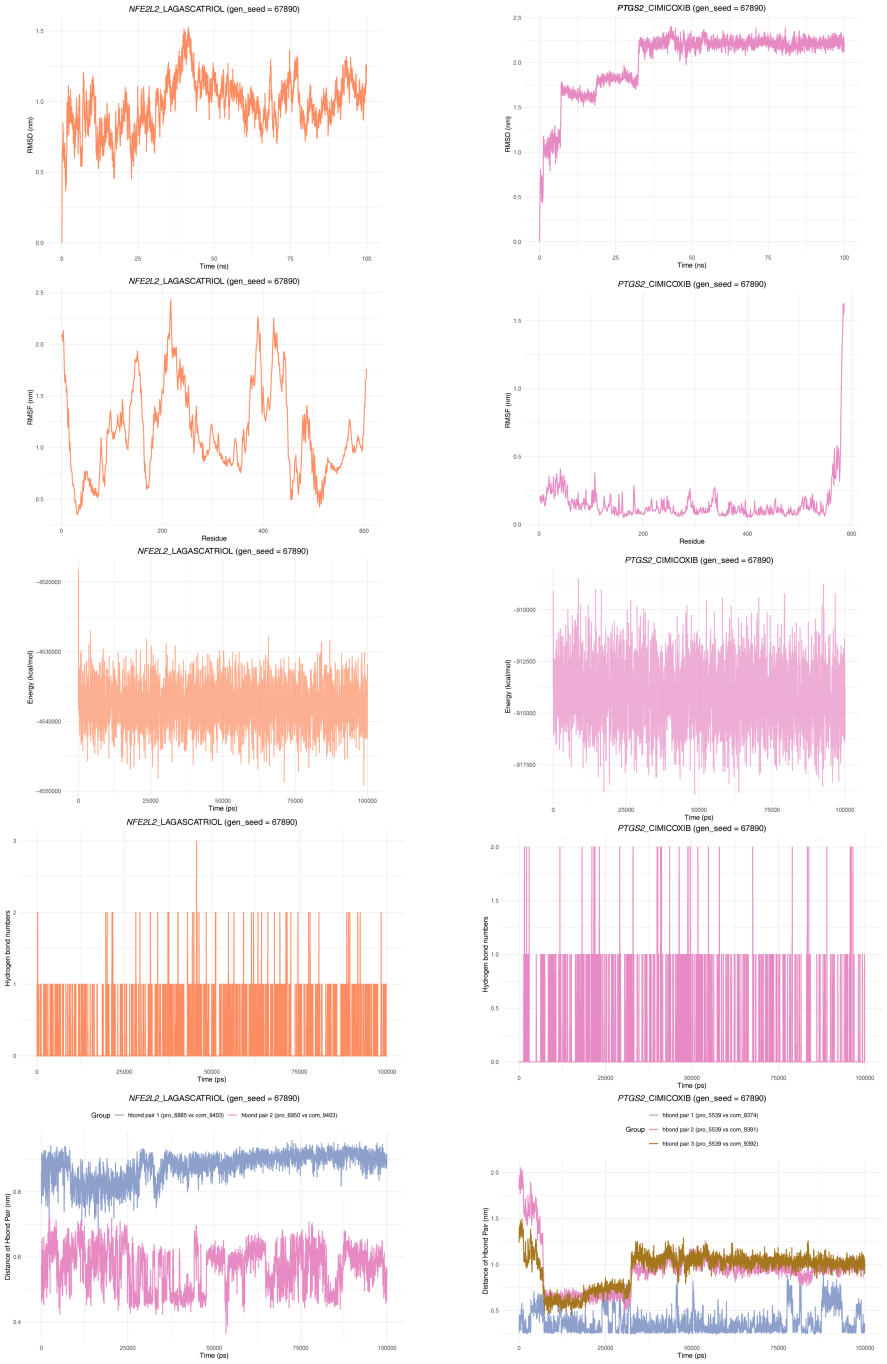
Reproducibility of molecular dynamics simulation with gen_seed = 67,890 for *NFE2L2*-LAGASCATRIOL and *PTGS2*-CIMICOXIB complexes.

To ensure the reproducibility of the experimental results, a molecular dynamics simulation was performed again with an initial velocity seed of 67,890 (gen_seed = 67,890) (Fig. 10). The RMSD results showed that the *NFE2L2*-LAGASCATRIOL complex achieved a relatively stable dynamic equilibrium after initial conformational adjustment. The *PTGS2*-CIMICOXIB complex entered stable dynamic equilibrium after 30 ns, indicating system stability and stable ligand binding. The RMSF of *NFE2L2*-LAGASCATRIOL showed a “high at both ends with mid-region fluctuations” pattern, consistent with typical protein flexibility. *PTGS2*-CIMICOXIB maintained overall stability (low RMSF in most regions) with localized high-flexibility residues. Meanwhile, both complexes exhibited stable energy fluctuations, confirming the thermodynamic stability of the systems and ligand-protein interactions. Furthermore, *NFE2L2*-LAGASCATRIOL formed 1–2 hydrogen bonds, showing dynamic equilibrium with minor fluctuations. *PTGS2*-CIMICOXIB primarily formed 1 stable hydrogen bond, providing basic binding stability. *NFE2L2*-LAGASCATRIOL’s two hydrogen bond pairs showed stable distance fluctuations. *PTGS2*-CIMICOXIB’s hydrogen bond pairs maintained stable distances with small fluctuations.

### Experimental validation of *NFE2L2* and *PTGS2*

**Figure 11:**
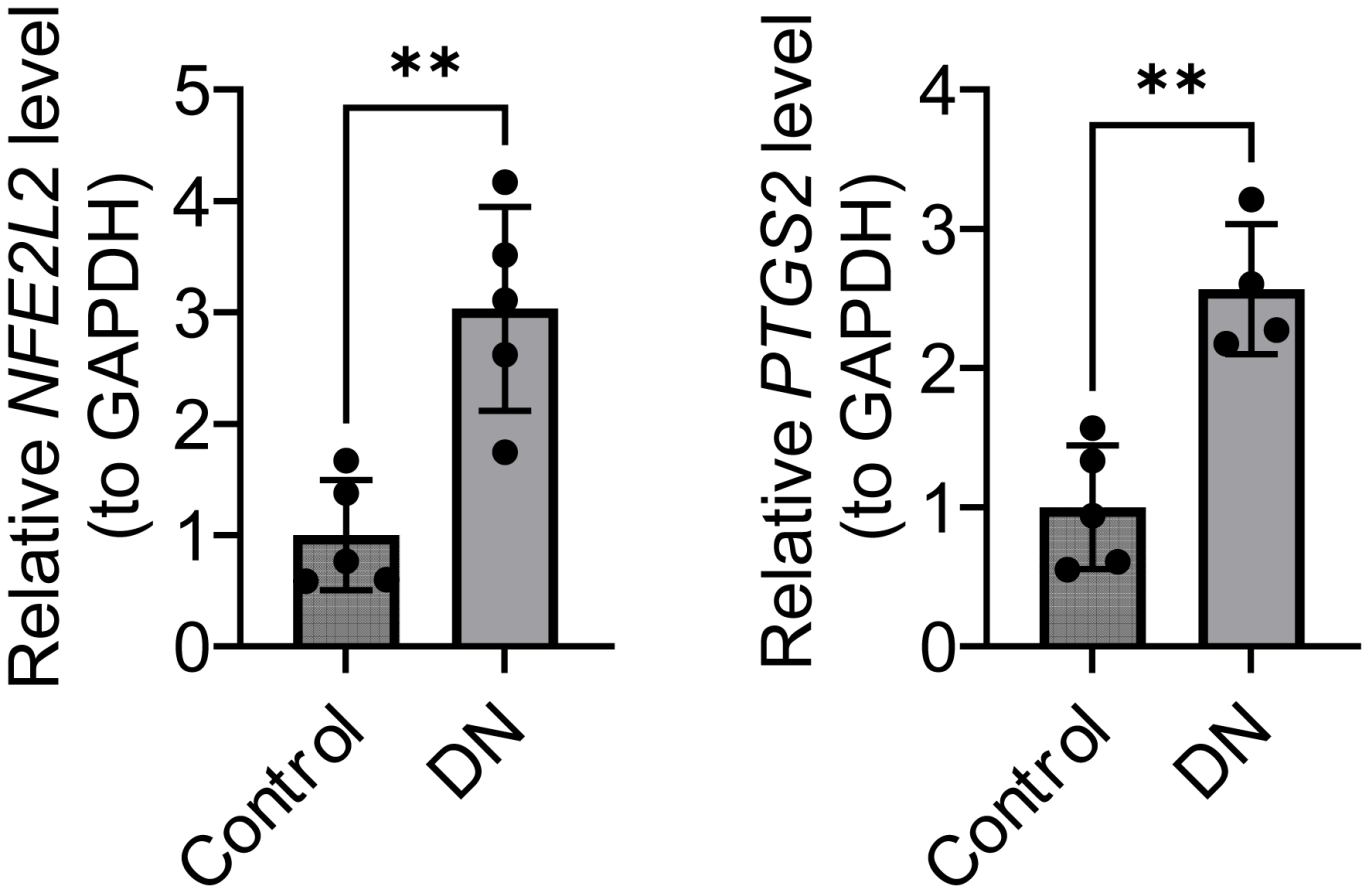
Relative expression levels of *NFE2L2* and *PTGS2* (normalized to GAPDH). *p < 0.01.

The expression of *NFE2L2* and *PTGS2* was further analyzed on clinical samples using RT-qPCR. *NFE2L2* and *PTGS2* were significantly higher in DN samples compared with those in the control group (*p* < 0.05) (Fig. 11). The results of the clinical trial were consistent with those of the bioinformatics analysis, which strengthens the reliability of this bioinformatics analysis.

## Discussion

DN is a leading cause of end-stage kidney disease, involving dysregulated metabolic pathways such as lipid metabolism, glycolysis, and enhanced lactate metabolism (Fujisawa et al., 2023). Studies show reduced serum lactate levels in DN models, indicating its homeostatic role (Dugan et al., 2013). Beyond metabolism, lactate acts as a signaling molecule, regulating downstream pathways and post-translational modifications, impacting glucose/lipid metabolism (Gong et al., 2024; Yang et al., 2023b). These insights position lactate as an active contributor to DN progression, making the identification of key lactate regulators vital for understanding mechanisms and developing therapies.

Bioinformatic analyses identified two lactate metabolism-related biomarkers, *PTGS2* and *NFE2L2*. Further analyses, including GSEA, GSVA, immune infiltration, drug prediction, and molecular docking, clarified their roles and mechanisms.

*PTGS2*, also known as COX-2, is a key enzyme in prostaglandin synthesis, promoting PGE_2_ production, which facilitates immune cell infiltration and activation in the kidney (Martín-Vázquez et al., 2023; Huang et al., 2020). Activated immune cells, especially M1 macrophages, exhibit enhanced glycolysis and lactate secretion, akin to the Warburg effect in cancer, driven by inflammatory signals (Yang et al., 2023a; Kim et al., 2025). PTGS2 upregulation may amplify inflammation and create a microenvironment conducive to lactate overproduction, regulated by factors like HIF-1α, involved in diabetic nephropathy (Hu et al., 2024; Huang et al., 2016). Both inflammation and lactate metabolism are major drivers of DN progression (Guan et al., 2025). Our findings show increased PTGS2 in diabetic nephropathy, suggesting it enhances inflammation, influences immune cell metabolism, promotes glycolysis, and exacerbates DN. *NFE2L2*/NRF2 is a key regulator of cellular antioxidant defenses, activating target genes to neutralize reactive oxygen species (ROS) and protect mitochondria from oxidative damage (Li et al., 2021; Matana et al., 2020). Impaired NRF2 function reduces mitochondrial oxidative phosphorylation efficiency, leading to decreased lactate metabolism and accumulation (Piantadosi et al., 2008; Xiao et al., 2020). These findings suggest NRF2’s role in maintaining mitochondrial redox balance and intracellular lactate homeostasis. In DN, RT-qPCR shows increased *NFE2L2* mRNA, possibly as a compensatory response to hyperglycemia and oxidative stress. However, despite elevated expression, NRF2 activity may be insufficient to counteract oxidative damage fully. This functional inadequacy may worsen mitochondrial dysfunction, impair lactate clearance, and accelerate DN progression.

The aforementioned findings suggest that *PTGS2* and *NFE2L2* may participate in the reprogramming of lactate metabolism in DN through the inflammatory and oxidative stress pathways, respectively. The inflammatory response mediated by *PTGS2* promotes lactate production, while the antioxidant pathway regulated by *NFE2L2* modulates lactate clearance. The imbalance between these two processes jointly leads to lactate accumulation in the renal tissue microenvironment, resulting in lactate metabolic disorders and further accelerating the progression of DN. However, whether and how *PTGS2* and *NFE2L2* directly regulate the expression or activity of key enzymes involved in lactate metabolism remains to be verified by subsequent functional experiments using cellular and animal models. In the future, in-depth clarification of the exact roles of these two genes in DN-associated lactate metabolic disorders will provide an important theoretical basis for the development of novel therapeutic strategies targeting the metabolism-immunity-oxidative stress network. Based on GSEA, lactate metabolism-associated genes in DN are significantly enriched in primary immunodeficiency, p53 signaling pathway, and cytokine-cytokine receptor interaction pathways. Primary immunodeficiency pathway enrichment indicates immune dysfunction in DN progression. Diabetes-associated immune impairment with elevated inflammatory mediators (Wang et al., 2021) may exacerbate renal damage through increased infection susceptibility and autoimmune reactions (Zhang, Liang & Liu, 2024). Antibody deficiency-related immune dysregulation associates with DN development (Šedivá et al., 2021), particularly in young patients with recurrent infections and glomerular lesions (Caza, Hassen & Larsen, 2020). Activated immune cells utilize lactate to sustain functions, and immune activation further promotes lactate accumulation, forming a feedback loop that may worsen DN (Ye, Jiang & Zhang, 2022). Altered immune cell metabolism can increase lactate production, which in turn modulates immune responses, potentially accelerating disease progression. The p53 pathway plays crucial roles in DN pathogenesis. Hyperglycemia-induced p53 upregulation regulates apoptosis, inflammation, and fibrosis (Liang et al., 2021; Ma et al., 2019), while also modulating mitochondrial function and energy metabolism balance (Matoba et al., 2006). p53-mediated mitochondrial dysfunction and impaired autophagy exacerbate renal injury, with miR-155-5p interaction affecting Sirt1 expression and fibrosis (Wang et al., 2018).

Cytokine-cytokine receptor interaction enrichment emphasizes immune-inflammatory significance in DN. Multiple cytokines mediate immune and inflammatory processes in nephropathy progression (Cao et al., 2020), with TGF-β and connective tissue growth factor being particularly important (Jia et al., 2020). Therapeutic targeting of these pathways shows potential, as demonstrated by asiatic acid’s inhibition of TGF-βR1 (Zhang et al., 2022). Overall, lactate metabolism-related genes influence DN by integrating the interactive network of immune regulation, cellular stress, and inflammation, offering promising avenues for mechanistic research.

Immunocorrelation analysis indicates that biomarkers modulate immune cell activity, influencing DN progression and therapy response. Understanding these interactions could guide immunotherapeutic development and personalized treatment strategies for DN. Evidence highlights immune mechanisms in DN progression to ESRD, notably increased T follicular helper (Tfh) cell infiltration, consistent with findings by Qiu et al. (2023) and Zhang et al. (2016), suggesting Tfh cells as potential intervention targets. Immune cell infiltration, including memory B cells, is elevated in DN tissues compared to controls, with Li et al. (2022b) reporting increased activated dendritic cells, M1 macrophages, and B cells in glomeruli.

CD8+ T cells, especially tissue-resident subsets, are increased in DN, contributing to podocyte injury and glomerular sclerosis *via* IL-15 activation (Li et al., 2022a). Effector memory CD8+ T cells may also promote renal inflammation through miR-186-5p secretion (Xu et al., 2023). These cells are potential therapeutic targets for mitigating immune-mediated renal damage.

Cimicoxib, a selective COX-2 inhibitor, is used to treat depression and schizophrenia (Correa-Rotter et al., 2024). If approved, it would be the first novel mechanism drug for depression in decades. Both disorders involve inflammatory processes, with elevated pro-inflammatory cytokines and PGE levels reported in major depression (Perry et al., 2021; Davis et al., 2022). COX-2 inhibitors like Cimicoxib may have antidepressant effects *via* anti-inflammatory action (Gumułka et al., 2023). Further research is needed to confirm its efficacy in DN.

RT-qPCR validated the predicted expression of *NFE2L2* and *PTGS2* in DN patients, supporting the reliability of the database and current findings (Chen et al., 2023; Wang et al., 2022).

This study faces several limitations. The results depend on public database quality and analysis assumptions, risking data noise and false results. The validation cohort was small and lacked detailed disease staging, limiting analysis of *PTGS2* and *NFE2L2* expression across disease stages and their early diagnostic potential. Although identified as key biomarkers, lactate levels were not measured, preventing direct gene-metabolism correlation. Subcellular localization was predicted without experimental validation. Functional mechanisms were only preliminarily confirmed *via* RT-qPCR, without gene knockdown or overexpression studies to clarify their roles in lactate metabolism and renal injury. Future work will expand sample size, include more validation cohorts, and systematically gather disease staging data. We will use DN animal models at different stages to track biomarker changes, complemented by *in vitro* high-glucose experiments on renal tubular cells to study disease progression. This aims to evaluate *PTGS2* and *NFE2L2* as early diagnostic markers. Additional methods include lactate measurement, immunofluorescence, and functional gene experiments in cells and animals, supported by molecular biology validations to strengthen diagnostic and therapeutic insights for DN.

## Conclusions

In our study, biomarkers related to lactate metabolism in DN were identified and validated through the above bioinformatics analysis. It has been demonstrated that *NFE2L2* has protective and therapeutic potential in DN rats. The above bioinformatics analysis provides a solid theoretical basis and experimental guidance for an in-depth understanding of the pathogenesis and diagnostic model of DN, and provides an important reference for future clinical practice and basic research.

## Supplemental Information

10.7717/peerj.20761/supp-1 Supplemental Information 1 MIQE checklist

10.7717/peerj.20761/supp-2 Supplemental Information 2 RMSF of molecules during simulation. RMSF quantifies the dynamic flexibility of atomic positions, with peak regions typically corresponding to higher conformational freedom

10.7717/peerj.20761/supp-3 Supplemental Information 3 The variation in RMSD over time during the simulation process RMSD serves to quantify the deviation of a structure relative to its reference conformation, and its convergence can be used to assess the stability of the simulation.

10.7717/peerj.20761/supp-4 Supplemental Information 4 The variation in hydrogen bond count over time during the simulation The hydrogen bond count serves as a key indicator for evaluating intermolecular bonding strength and the structural stability of the system.

10.7717/peerj.20761/supp-5 Supplemental Information 5 Distribution of distances between site 554 and associated sites/molecules This distance information may be utilised to analyse the site’s specific interactions and their spatial conformation relationships.

10.7717/peerj.20761/supp-6 Supplemental Information 6 The variation of total energy over time within the simulated system Total energy encompasses both kinetic and potential energy. By observing changes in total energy, one can assess whether the simulation has reached a state of equilibrium and evaluate the system’s energy stability.

10.7717/peerj.20761/supp-7 Supplemental Information 7 NFE2L2_LAGASCATRIOL Structural file of the molecular docking complex between NFE2L2 and LAGASCATRIOL.

10.7717/peerj.20761/supp-8 Supplemental Information 8 Three-dimensional structure of CIMICOXIB

10.7717/peerj.20761/supp-9 Supplemental Information 9 Three-dimensional structure of PTGS2

10.7717/peerj.20761/supp-10 Supplemental Information 10 The fluctuation of each atom in the molecule relative to its average position

10.7717/peerj.20761/supp-11 Supplemental Information 11 Velocity-related information between atomic groups of CIMICOXIB and PTGS2, recording the trend of the distance between atomic group 3 and atomic group 6 during the simulation

10.7717/peerj.20761/supp-12 Supplemental Information 12 Distance distribution information related to molecular parts

10.7717/peerj.20761/supp-13 Supplemental Information 13 The variation of total energy in the simulation system over time

10.7717/peerj.20761/supp-14 Supplemental Information 14 Velocity-related information between atomic groups of CIMICOXIB and PTGS2, recording the trend of the distance between atomic group 6 and atomic group 2 during the simulation

10.7717/peerj.20761/supp-15 Supplemental Information 15 Velocity-related information between atomic groups of CIMICOXIB and PTGS2, recording the trend of the distance between atomic group 6 and atomic group 5 during the simulation

10.7717/peerj.20761/supp-16 Supplemental Information 16 Velocity-related distance changes between atomic group 6 and atomic group 3 under the original speed simulation

10.7717/peerj.20761/supp-17 Supplemental Information 17 The degree of deviation of the molecular structure from the reference structure during the simulation

10.7717/peerj.20761/supp-18 Supplemental Information 18 Records distance information between site number 276 and other related sites or molecular parts

10.7717/peerj.20761/supp-19 Supplemental Information 19 Velocity-related information between atomic groups of CIMICOXIB and PTGS2, recording the trend of the distance between atomic group 6 and atomic group 1 during the simulation

10.7717/peerj.20761/supp-20 Supplemental Information 20 The variation in the number of hydrogen bonds during the simulation over time

10.7717/peerj.20761/supp-21 Supplemental Information 21 The results of the last frame in molecular dynamics simulation

10.7717/peerj.20761/supp-22 Supplemental Information 22 Velocity-related information between atomic groups of CIMICOXIB and PTGS2, recording the trend of the distance between atomic group 2 and atomic group 26 during the simulation

10.7717/peerj.20761/supp-23 Supplemental Information 23 Velocity-related information between atomic groups of CIMICOXIB and PTGS2, recording the trend of the distance between atomic group 3 and atomic group 24 during the simulation

10.7717/peerj.20761/supp-24 Supplemental Information 24 Velocity-related information between atomic groups of CIMICOXIB and PTGS2, recording the trend of the distance between atomic group 3 and atomic group 25 during the simulation

10.7717/peerj.20761/supp-25 Supplemental Information 25 Velocity-related information between atomic groups of CIMICOXIB and PTGS2, recording the trend of the distance between atomic group 5 and atomic group 23 during the simulation

10.7717/peerj.20761/supp-26 Supplemental Information 26 Velocity-related information between atomic groups of CIMICOXIB and PTGS2, recording the trend of the distance between atomic group 10 and atomic group 23 during the simulation

10.7717/peerj.20761/supp-27 Supplemental Information 27 Velocity-related information between atomic groups of CIMICOXIB and PTGS2, recording the trend of the distance between atomic group 10 and atomic group 25 during the simulation

10.7717/peerj.20761/supp-28 Supplemental Information 28 Velocity-related information between atomic groups of CIMICOXIB and PTGS2, recording the trend of the distance between atomic group 12 and atomic group 25 during the simulation

10.7717/peerj.20761/supp-29 Supplemental Information 29 Velocity-related information between atomic groups of CIMICOXIB and PTGS2, recording the trend of the distance between atomic group 17 and atomic group 27 during the simulation

10.7717/peerj.20761/supp-30 Supplemental Information 30 Velocity-related information between atomic groups of CIMICOXIB and PTGS2, recording the trend of the distance between atomic group 24 and atomic group 1 during the simulation

10.7717/peerj.20761/supp-31 Supplemental Information 31 Velocity-related information between atomic groups of CIMICOXIB and PTGS2, recording the trend of the distance between atomic group 24 and atomic group 4 during the simulation

10.7717/peerj.20761/supp-32 Supplemental Information 32 Velocity-related information between atomic groups of CIMICOXIB and PTGS2, recording the trend of the distance between atomic group 42 and atomic group 9 during the simulation

10.7717/peerj.20761/supp-33 Supplemental Information 33 Velocity-related information between atomic groups of CIMICOXIB and PTGS2, recording the trend of the distance between atomic group 24 and atomic group 15 during the simulation

10.7717/peerj.20761/supp-34 Supplemental Information 34 Velocity-related information between atomic groups of CIMICOXIB and PTGS2, recording the trend of the distance between atomic group 12 and atomic group 24 during the simulation

10.7717/peerj.20761/supp-35 Supplemental Information 35 Velocity-related information between atomic groups of CIMICOXIB and PTGS2, recording the trend of the distance between atomic group 20 and atomic group 23 during the simulation

10.7717/peerj.20761/supp-36 Supplemental Information 36 Velocity-related information between atomic groups of CIMICOXIB and PTGS2, recording the trend of the distance between atomic group 2 and atomic group 24 during the simulation

10.7717/peerj.20761/supp-37 Supplemental Information 37 Velocity-related information between atomic groups of CIMICOXIB and PTGS2, recording the trend of the distance between atomic group 19 and atomic group 23 during the simulation

10.7717/peerj.20761/supp-38 Supplemental Information 38 Velocity-related information between atomic groups of CIMICOXIB and PTGS2, recording the trend of the distance between atomic group 4 and atomic group 26 during the simulation

10.7717/peerj.20761/supp-39 Supplemental Information 39 Velocity-related information between atomic groups of CIMICOXIB and PTGS2, recording the trend of the distance between atomic group 12 and atomic group 26 during the simulation

10.7717/peerj.20761/supp-40 Supplemental Information 40 Velocity-related information between atomic groups of CIMICOXIB and PTGS2, recording the trend of the distance between atomic group 19 and atomic group 22 during the simulation

10.7717/peerj.20761/supp-41 Supplemental Information 41 Velocity-related information between atomic groups of CIMICOXIB and PTGS2, recording the trend of the distance between atomic group 24 and atomic group 6 during the simulation

10.7717/peerj.20761/supp-42 Supplemental Information 42 Velocity-related information between atomic groups of CIMICOXIB and PTGS2, recording the trend of the distance between atomic group 24 and atomic group 7 during the simulation

10.7717/peerj.20761/supp-43 Supplemental Information 43 Velocity-related information between atomic groups of CIMICOXIB and PTGS2, recording the trend of the distance between atomic group 24 and atomic group 8 during the simulation

10.7717/peerj.20761/supp-44 Supplemental Information 44 Velocity-related information between atomic groups of CIMICOXIB and PTGS2, recording the trend of the distance between atomic group 24 and atomic group 10 during the simulation

10.7717/peerj.20761/supp-45 Supplemental Information 45 Velocity-related information between atomic groups of CIMICOXIB and PTGS2, recording the trend of the distance between atomic group 24 and atomic group 11 during the simulation

10.7717/peerj.20761/supp-46 Supplemental Information 46 Velocity-related information between atomic groups of CIMICOXIB and PTGS2, recording the trend of the distance between atomic group 24 and atomic group 13 during the simulation

10.7717/peerj.20761/supp-47 Supplemental Information 47 Velocity-related information between atomic groups of CIMICOXIB and PTGS2, recording the trend of the distance between atomic group 24 and atomic group 14 during the simulation

10.7717/peerj.20761/supp-48 Supplemental Information 48 Velocity-related information between atomic groups of CIMICOXIB and PTGS2, recording the trend of the distance between atomic group 24 and atomic group 16 during the simulation

10.7717/peerj.20761/supp-49 Supplemental Information 49 Velocity-related information between atomic groups of CIMICOXIB and PTGS2, recording the trend of the distance between atomic group 24 and atomic group 18 during the simulation

10.7717/peerj.20761/supp-50 Supplemental Information 50 Velocity-related information between atomic groups of CIMICOXIB and PTGS2, recording the trend of the distance between atomic group 24 and atomic group 19 during the simulation

10.7717/peerj.20761/supp-51 Supplemental Information 51 Velocity-related information between atomic groups of CIMICOXIB and PTGS2, recording the trend of the distance between atomic group 24 and atomic group 21 during the simulation

10.7717/peerj.20761/supp-52 Supplemental Information 52 Changes in the number of hydrogen bonds during the simulation of CIMICOXIB and PTGS2 over time

10.7717/peerj.20761/supp-53 Supplemental Information 53 Changes in the total energy of the CIMICOXIB-PTGS2 simulation system over time

10.7717/peerj.20761/supp-54 Supplemental Information 54 Velocity-related information between atomic groups of CIMICOXIB and PTGS2, recording the trend of the distance between atomic group 19 and atomic group 25 during the simulation

10.7717/peerj.20761/supp-55 Supplemental Information 55 Velocity-related information between atomic groups of CIMICOXIB and PTGS2, recording the trend of the distance between atomic group 3 and atomic group 26 during the simulation

10.7717/peerj.20761/supp-56 Supplemental Information 56 The results of the last frame in molecular dynamics simulation

10.7717/peerj.20761/supp-57 Supplemental Information 57 RMSF of CIMICOXIB and PTGS2, reflecting the fluctuation of each atom in the molecule relative to its average position

10.7717/peerj.20761/supp-58 Supplemental Information 58 RMSD of CIMICOXIB and PTGS2, used to measure the deviation of molecular structures from the reference structure during the simulation

10.7717/peerj.20761/supp-59 Supplemental Information 59 Code for molecular docking between NFE2L2 and LAGASCATRIOL

10.7717/peerj.20761/supp-60 Supplemental Information 60 Lactate metabolism related genes

10.7717/peerj.20761/supp-61 Supplemental Information 61 General clinical characteristics in DN patients and healthy participants

10.7717/peerj.20761/supp-62 Supplemental Information 62 List of candidate genes

10.7717/peerj.20761/supp-63 Supplemental Information 63 The enriched termes of gene ontology (GO) analysis

10.7717/peerj.20761/supp-64 Supplemental Information 64 The enriched pathways of kyoto encyclopedia of genes and genomes (KEGG) analysis

10.7717/peerj.20761/supp-65 Supplemental Information 65 The kyoto encyclopedia of genes and genomes (KEGG) pathways of PTGS2 in gene set enrichment analysis (GSEA) analysis

10.7717/peerj.20761/supp-66 Supplemental Information 66 The kyoto encyclopedia of genes and genomes (KEGG) pathways of NFE2L2 in gene set enrichment analysis (GSEA) analysis

10.7717/peerj.20761/supp-67 Supplemental Information 67 The correlation between different immune cells

10.7717/peerj.20761/supp-68 Supplemental Information 68 The miRNAs linked to PTGS2

10.7717/peerj.20761/supp-69 Supplemental Information 69 The RBPs linked to biomarkers

10.7717/peerj.20761/supp-70 Supplemental Information 70 The disease types related to PTGS2

10.7717/peerj.20761/supp-71 Supplemental Information 71 The disease types related to NFE2L2

10.7717/peerj.20761/supp-72 Supplemental Information 72 The interaction score of drugs

10.7717/peerj.20761/supp-73 Supplemental Information 73 PR curve analysis results

## Additional Information and Declarations

### Data Availability

The following information was supplied regarding data availability: The raw data are available in the Supplemental Files and figshare: zhang, xiaojuan (2025). Biomarkers in Diabetic Nephropathy (DN). figshare. Figure. https://doi.org/10.6084/m9.figshare.30484325.v1. The Supplementary Files include information related to genes, immune cells, miRNAs, diseases, drugs, *etc.*, which can help to further understand lactic acid metabolism, immune responses, and the biological functions of specific genes and their associations with diseases. The Supplementary Files are in XVG format, which is the original output file of protein–protein interaction network data/differential expression gene enrichment analysis results. The required reading software is VMD, which can be obtained for free or used with compliant authorization through the official website (https://www.ks.uiuc.edu/Research/vmd/).
