# Supplementary figures and images for: Identification and validation of biomarkers associated with lactic acid metabolism in diabetic nephropathy
Source: PeerJ. 2026 Mar 3;14:e20761. doi: 10.7717/peerj.20761 (PMC12965171; doi:10.7717/peerj.20761)

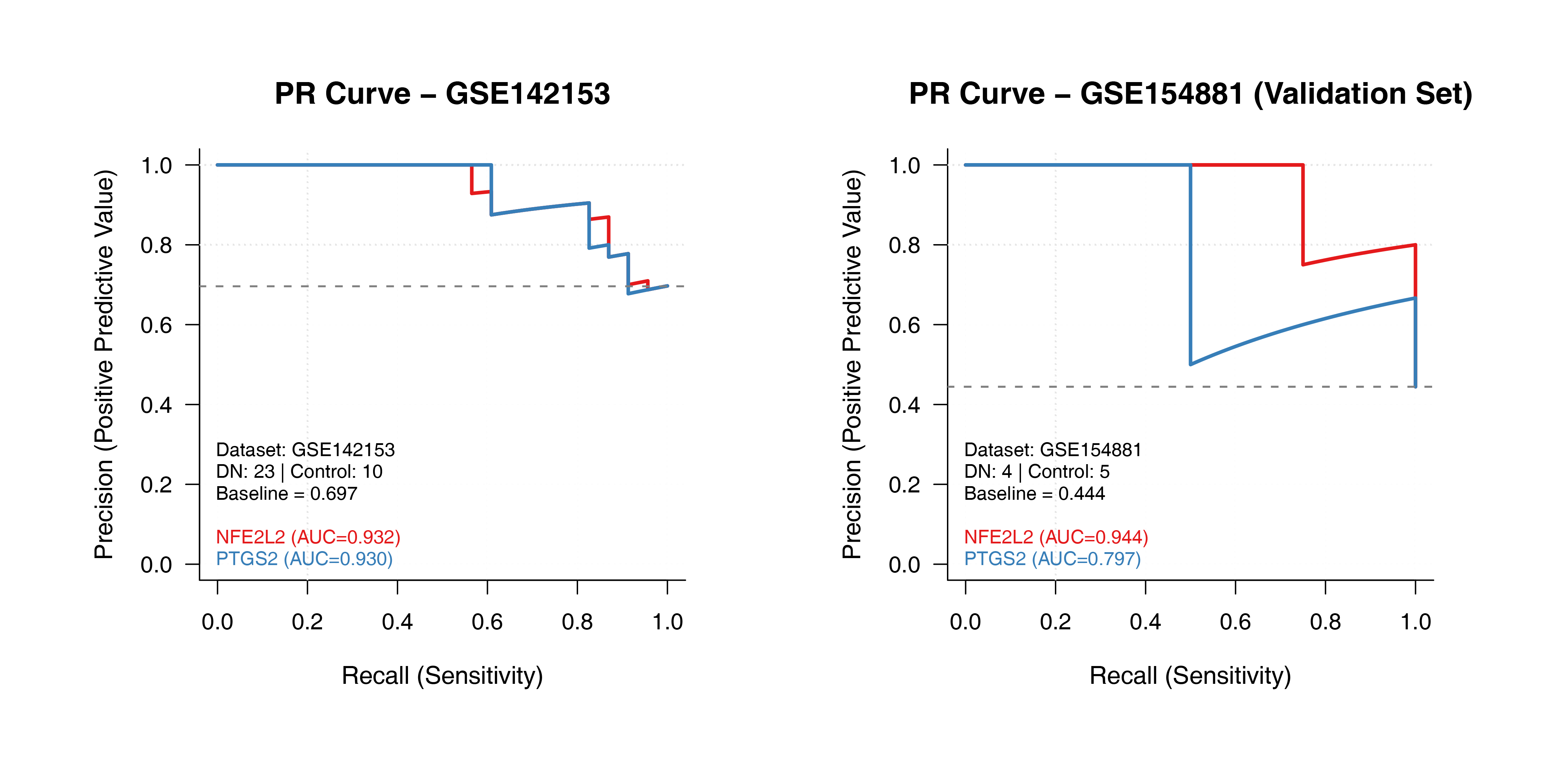

Supplement: Supplemental Information 73 [file peerj-14-20761-s073.png]
